# *Epimedium*: A Versatile Player in Bone Tissue Engineering

**DOI:** 10.3390/ijms27219579

**Published:** 2026-10-26

**Authors:** Tianyou Xu, Rao Zhang, Jiaxing Hu, Junnan Jiang, Longjie Qiao, Sijie Deng, Xinyang Li, Wenjun Zhang, Zhengxin Zhou, Kui Xu, Caiyun Mu

**Affiliations:** 1 College of Acumox and Tuina (College of Rehabilitation Medicine), Anhui University of Chinese Medicine, Hefei 230012, China; 2021205315043@stu.ahtcm.edu.cn (T.X.);; 2 The First Clinical Medical School, Anhui University of Chinese Medicine, Hefei 230038, Chinazhouzhengxin1968@sina.com (Z.Z.); 3 School of Integrated Chinese and Western Medicine, Anhui University of Chinese Medicine, Hefei 230012, China; 4 College of Medical Information Engineering, Anhui University of Chinese Medicine, Hefei 230012, China; wenjun@ahtcm.edu.cn

**Keywords:** *Epimedium*, bioactive constituents, bone tissue engineering, bone-related biological functions, network pharmacology

## Abstract

*Epimedium* is a traditional herb that has been investigated for its potential osteogenic and bone-regenerative effects, yet systematic analyses of its multi-component, multi-target mechanisms in bone metabolism and tissue engineering applications are limited. To address this knowledge gap, this narrative review systematically summarizes more than 20 bioactive constituents extracted from various parts of major medicinal *Epimedium* species. We elaborate on their pivotal roles in modulating osteogenic differentiation, suppressing osteoclastogenesis and bone resorption, regulating inflammatory responses, exerting antioxidant effects, promoting angiogenesis, demonstrating antitumor activity, exerting chondroprotective effects, and exhibiting antibacterial activity during bone defect repair. Furthermore, we highlight recent advancements and current trends in applying these bioactive constituents within the field of bone tissue engineering. Additionally, we compared the osteogenic and osteoprotective potentials of three metabolically related flavonoids (icariin, icaritin, and icariside II). Network pharmacology was utilized to elucidate their multi-target mechanisms in modulating bone metabolism, providing insights for future mechanistic studies. Despite the long-standing clinical use and extensive scientific investigation of *Epimedium*, this review identifies several challenges associated with its application in bone tissue engineering and offers constructive recommendations to overcome these limitations.

## 1. Introduction

Bone, a marvel of nature’s engineering, is a dynamic and highly vascularized tissue that undergoes continuous remodeling throughout an individual’s lifetime. Although bone possesses a robust regenerative capacity, severe bone injuries and pathological conditions often fail to heal spontaneously due to the lack of structural scaffolds for new bone formation and positive regulatory signals required for bone regeneration [1]. Traditional autologous or allogeneic bone grafts present clinical limitations, such as donor site morbidity, restricted availability, and potential long-term complications, thereby urgently necessitating advanced bone tissue engineering solutions [2].

Successful bone regeneration is a highly complex process involving the regulation of osteogenic and osteoclastic differentiation, inflammatory responses, oxidative stress, and angiogenesis. Among these, the coordinated regulation of osteogenic differentiation and the suppression of osteoclastogenesis constitute the core mechanism for maintaining bone metabolic homeostasis in bone tissue engineering. A key challenge in current research is the development of biomaterial-based delivery systems to achieve targeted inhibition of osteoclast activity while preserving normal physiological bone remodeling processes [3,4]. Because chronic inflammation and oxidative stress can impair osteoblast function and hyperactivate osteoclasts, developing scaffold materials with integrated antioxidant and anti-inflammatory properties to modulate the local tissue microenvironment has emerged as a major challenge [3,4,5,6]. Angiogenesis supports osteogenesis by providing essential nutrients and signaling molecules, such as vascular endothelial growth factor (VEGF) secreted by endothelial cells [5,6,7]. However, the architectural design of vascular channels within scaffolds must balance adequate mechanical strength with optimal biological performance. Furthermore, treatments for specific clinical conditions impose additional requirements on ideal scaffolds. For instance, following bone tumor resections, implants (e.g., titanium (Ti) and its alloys, polyetheretherketone) must simultaneously release anticancer drugs to inhibit tumor recurrence and promote rapid osteogenesis [8,9,10]. For osteochondral interface defects, scaffold design should focus on the unique biomechanical properties of cartilage (e.g., a low friction coefficient and high compressive resilience) and its avascular microenvironment to effectively repair the bone–cartilage junction [11]. Finally, implant materials must overcome bacterial biofilm formation and local microenvironmental alterations caused by infections (such as hypoxia and acidic pH). Thus, antibacterial properties represent a critical factor in ensuring successful bone regeneration.

*Epimedium*, recognized as a potent promoter of bone healing [12], was first documented in the Shennong’s Herbal Classic (circa 200–300 AD). In the Compendium of Materia Medica, compiled by Shizhen Li during the Ming Dynasty, *Epimedium* was described in detail for its therapeutic properties, including strengthening muscles and bones, enhancing vital energy, benefiting the liver and kidneys, and improving mental clarity. Modern pharmacological studies further support these traditional claims, demonstrating that multiple bioactive constituents of *Epimedium* exhibit significant bone-protective effects [13,14,15,16,17]. Due to its diverse chemical composition and broad pharmacological activities, *Epimedium* has become a focal point in the research of medicinal plants and their applications in treating various diseases. Overall, in the context of bone tissue engineering, *Epimedium* exerts its pharmacological effects through multiple biological pathways. With respect to bone metabolism, its active constituents promote the differentiation of mesenchymal stem cells into osteoblasts and modulate key signaling pathways to suppress osteoclast activity, thereby enhancing the balance between bone formation and resorption [18,19]. The antioxidant properties are characterized by the scavenging of free radicals and the upregulation of endogenous antioxidant enzyme activities, which mitigate oxidative stress-induced cellular damage [20,21]. Its anti-inflammatory effects are mediated through the inhibition of pro-inflammatory signaling cascades and the regulation of immune factor secretion [22,23,24]. The anti-tumor activity involves the induction of tumor cell apoptosis, suppression of angiogenesis, and enhancement of immune surveillance [25,26,27,28]. Furthermore, icariin, a major flavonoid of *Epimedium*, induces endothelium-dependent vasorelaxation and improves vascular endothelial function, effects that may promote local perfusion and support vascularized bone regeneration [29,30]. This multi-target, multi-pathway mechanism of action positions *Epimedium* as a bridging agent between traditional medicine and modern bone repair technologies, thereby opening new avenues for research in the clinical management of complex bone defects. Furthermore, its antibacterial properties and chondroprotective effects contribute to establishing a favorable microenvironment throughout the entire bone defect repair process [31,32].

Based on the aforementioned rationales, this narrative review aims to inspire researchers to explore further potential applications of *Epimedium* in bone tissue engineering, by artfully synthesizing the latest advancements in elucidating its bioactive compounds’ regulatory mechanisms. The review focuses on analyzing over twenty bioactive compounds extracted from key medicinal *Epimedium* species (including *Epimedium brevicornum* Maxim., *Epimedium sagittatum* Maxim., *Epimedium pubescens* Maxim., *Epimedium sutchuenense* Franch., *Epimedium wushanense* T. S. Ying, *Epimedium acuminatum* Franch., and *Epimedium koreanum* Nakai.) to explore their intricate roles in bone defect repair. We elucidate their diverse biological activities across multiple targets and highlight the advanced drug delivery systems engineered to enhance therapeutic potency. Flavonoids are the most widely utilized active constituents derived from *Epimedium*. Notably, icariin, icariside II, and icaritin exhibit a well-defined metabolic conversion relationship [14,23]. However, ongoing debates regarding their physicochemical properties and relative bone-protective activity have hindered a comprehensive elucidation of the pharmacological substance basis of *Epimedium*. In closing, we reflect on prevailing challenges and chart a visionary course for future research, encompassing the convergence of multi-omics technologies, the design of intelligent biomaterials, artificial intelligence, and the development of sophisticated preclinical models, aiming to accelerate research progress and clinical translation in bone regeneration derived from *Epimedium* (Figure 1).

## 2. Active Constituents of *Epimedium*

The chemical investigation of *Epimedium* commenced in 1935, initially pioneered by the Japanese scholar Akai [33]. To date, more than 270 constituents have been isolated and identified from *Epimedium*, including flavonoids, polysaccharides, alkaloids, organic acids, phytosterols, and terpenoids [34]. These constituents collectively constitute the complex and diverse pharmacological basis of *Epimedium*, thereby providing a scientific rationale for its application in traditional Chinese medicine (Figure 2 and Table 1).

### 2.1. Flavonoids

Flavonoids are a common class of plant phenolic compounds, which can be categorized into bioflavonoids (e.g., vitamin P), isoflavones (e.g., phytoestrogens), and neoflavonoids (e.g., leucoanthocyanidins) [35]. Flavonoids represent the primary bioactive constituents and function as characteristic chemotaxonomic markers for the genus *Epimedium* [36]. The compounds constitute approximately 1% to 8% of the dry weight of *Epimedium* [37]. *Epimedium* contains a variety of 8-prenylflavonoids, such as icariin, icaritin, icariside I, icariside II (also known as baohuoside I), sagittatoside A, sagittatoside B, icaritin dirhamnoside, epimedin A, epimedin B, and epimedin C [38,39]. Among these, icariin, icariside II, epimedin A, epimedin B, and epimedin C are recognized as the most abundant bioactive flavonoids, with icariin, epimedin A, epimedin B, and epimedin C collectively accounting for over 52% of total flavonoids in dried *Epimedium* [40]. Notably, icariin and icariside II are regarded as the most promising bioactive components in *Epimedium* for their potential in bone metabolism regulation [41]. However, the concentrations of these bioactive components in *Epimedium* can vary significantly depending on the species, harvesting time, storage duration, and geographical origin. For instance, icariin predominates in *Epimedium brevicornum* Maxim. and *Epimedium koreanum* Nakai. varieties, followed by epimedin B, epimedin C, and epimedin A [40,42]. Conversely, in *Epimedium sagittatum* Maxim., *Epimedium pubescens* Maxim., and *Epimedium wushanense* T. S. Ying, epimedin C emerges as the most abundant constituent, succeeded by icariin, epimedin B, and epimedin A [40]. Quantitative analysis reveals the following mean proportions of icariin, epimedin A, epimedin B, and epimedin C among 15 investigated flavonoids: *Epimedium brevicornum* Maxim. (85.6%), *Epimedium koreanum* Nakai (82.3%), *Epimedium sagittatum* Maxim. (68.8%), *Epimedium pubescens* Maxim. (74.9%), and *Epimedium wushanense* T. S. Ying (69.8%) [40]. *Epimedium sagittatum* Maxim. demonstrates notably higher concentrations of epimedin C (10.88 ± 0.83 mg/g dry-weight basis (DWB)), icariin (11.21 ± 1.12 mg/g DWB), and icariside II (3.23 ± 0.24 mg/g DWB) compared with other *Epimedium* species, with these levels being influenced by geographical provenance [42]. *Epimedium pubescens* Maxim. is generally considered to possess the highest overall bioactive content [43]. Comparative studies under identical field conditions demonstrate that *Epimedium sagittatum* Maxim. exhibits significantly greater concentrations of key bioactive components (icariin, icariside II, and epimedin C) than *Epimedium pubescens* Maxim. when grown in equivalent abiotic and biotic environments [43].

#### 2.1.1. Principal Flavonoids

Icariin is the most abundant bioactive compound found in *Epimedium*, as supported by pharmacological research [44,45]. Structurally, icariin contains a glucose moiety attached at the C-3 position, a rhamnose group at the C-7 position, a methoxy group at C-4 position, and an isopentenyl group at C-8 position. The isopentenyl moiety at the C-8 position is believed to be the active component responsible for osteoblast differentiation [46]. Icaritin, a flavonoid aglycone derivative of icariin, is formed through the hydrolytic cleavage of glycosidic moieties from icariin, icariside I, and icariside II [47,48]. It serves as a major metabolite of icariin in both the intestine and serum [49,50,51,52,53]. Icariside is one of the bioactive constituents of *Epimedium*. It is demonstrated that following oral administration of icariin, various metabolites such as icariside I, icariside II, and icaritin are generated through degradation by intestinal flora [54].

Epimedin A, epimedin B, and epimedin C are flavonol glycosides and principal bioactive components of *Epimedium* [16,17,55]. Epimedin C is the most abundant flavonol glycoside among certain *Epimedium* species, such as *Epimedium brevicornum* Maxim. [55]. It serves as a key chemical marker for the quality control of *Epimedium* medicinal materials, with reported content ranging from 1.22 to 40.63 mg/g DWB [56,57]. Studies have indicated that other herbal components present in *Epimedium* extracts may reduce the oral bioavailability of epimedin C [58]. Moreover, Islam et al. investigated the relationship between the chemical fingerprint of *Epimedium* extracts and their estrogenic activity, revealing a strong correlation between the total content of epimedin A, epimedin B, epimedin C and the estrogenic activity of the extracts [59].

Hyperoside is also one of the common flavonoid components in *Epimedium*. Its content varies significantly depending on the plant species and processing methods [60,61]. It is also known as quercetin-3-O-β-D-galactoside pyran glycoside and belongs to the flavonol glycoside category [60,61]. The aglycone of hyperoside, quercetin, is the active component in plants. Astragalin is a major flavonoid compound in *Epimedium*. It has a great oral bioavailability (45%) and drug similarity (0.24), and is one of the important active components that enable it to exert anti-osteoporosis effects [62].

#### 2.1.2. Minor Flavonoids

Studies have quantified trace flavonoid aglycones present in *Epimedium* species, including apigenin, quercitrin, daidzein, luteolin, and kaempferol. These flavonoid aglycones exhibit an average concentration of 0.038 ± 0.0061 g per 100 g of ethanol extract. Apigenin, i.e., 4′,5,7-trihydroxyflavone, constitutes one of the minor flavonoids in *Epimedium*, with a concentration of 0.0062 ± 0.0019 g/100 g of ethanol extract [63]. The extraction of quercitrin (also known as quercetin-3-O-rhamnoside) from *Epimedium* yields only trace quantities, presenting significant challenges for its large-scale application. Daidzein is a flavonoid compound isolated from *Epimedium pubescens* Maxim, with relatively low concentrations [34]. Kaempferol is a trace flavonoid compound derived from *Epimedium brevicornum* Maxim, quantified at 0.0035 ± 0.0009 g per 100 g of ethanol extract [34]. Luteolin is a naturally occurring flavonoid extracted from *Epimedium sagittatum* Maxim. and *Epimedium koreanum* Nakai [34]. Compared with the flavonols such as quercetin or kaempferol, luteolin occurs at lower levels [64].

### 2.2. Polysaccharides

To date, over 20 types of polysaccharides have been isolated from *Epimedium*, which are primarily categorized into neutral polysaccharides (containing mannose and rhamnose) and acidic polysaccharides (containing uronic acids such as glucuronic acid) [65,66,67,68]. Polysaccharides purified from *Epimedium acuminatum* Franch. contain more than 50% xylose [68]. Notably, *Epimedium* polysaccharides enhance the solubility of flavonoids, such as icariin and icariside II, in a concentration-dependent manner, with acidic polysaccharides exhibiting a more pronounced solubilizing effect [69].

### 2.3. Alkaloids

To date, seven alkaloids have been isolated from plants of the genus *Epimedium* [34]. Among these compounds, epimediphine represents a novel alkaloid, magnoflorine is classified as a non-lipophilic alkaloid, and mortieridinone, chloramphenicol, beauvericin, (−)-4,6′-anhydrooxysporidinone, as well as the 4′-hydroxy derivative of (−)-oxysporidinone, are identified as amide-type alkaloids [34]. These compounds are characterized by complex ring structures and exhibit significant biological activities [70]. These alkaloids often act synergistically with flavonoids, such as epimedin, to modulate immune responses, inflammation, and oxidative stress [71]. The alkaloids present in *Epimedium* are predominantly composed of magnoflorine [34]. Magnoflorine is an isoquinoline alkaloid that has been proven to have significant anti-inflammatory effects.

### 2.4. Phenylpropanoids

Phenylpropanoids are a class of natural organic substances characterized by the phenylpropane skeleton (C6-C3), and their derivatives include simple phenylpropanoids, coumarins, lignans, and lignins [34]. Simple phenylpropanoids isolated from *Epimedium* species include chlorogenic acid, cryptochlorogenic acid, neochlorogenic acid, protocatechuic acid, and caffeic acid. Chlorogenic acid, an ester formed between caffeic acid and quinic acid, is present in the whole plants of *Epimedium* and occurs at relatively high concentrations in *Epimedium koreanum* Nakai [34]. Its caffeic acid moiety contains a benzene ring, a vinyl group, and two phenolic hydroxyl groups, structural features that confer potent biological activities.

Protocatechuic acid, chemically known as 3,4-dihydroxybenzoic acid, is a metabolite of anthocyanins. Structurally similar to gallic acid, caffeic acid, and vanillic acid, protocatechuic acid is a naturally occurring phenolic acid that is widely distributed in nature [72]. It has been isolated from *Epimedium koreanum* Nakai and is considered one of the key phenolic constituents in this plant species [73]. Caffeic acid, also referred to as 3,4-dihydroxycinnamic acid, is a phenolic compound commonly found in medicinal plants, coffee, and olive oil [74]. It has been identified as a significant aromatic organic acid in *Epimedium koreanum* Nakai and demonstrates notable anti-inflammatory and antioxidant effects [73,74,75]. Coumarin compounds feature a benzopyranone ring as their core structure, which is formed through the lactonization of o-hydroxycinnamic acid. Their defining structural characteristic is the fusion of a benzene ring with a pyranone ring. These compounds are distinguished by their low molecular weight, exceptional synthetic accessibility, and remarkable bioavailability [76]. However, the coumarin compounds in the *Epimedium* genus consist of only one type of compound, i.e., 5,7,4-trimethoxy-4-phenylcoumarin [34]. This compound is isolated from the leaves of *Epimedium brevicornum* Maxim. and its content is extremely low [34]. Although the quantity and diversity of coumarins in *Epimedium* are relatively limited, numerous other derivatives found in this genus have attracted significant research interest in the field of bone biology, highlighting their potential as promising therapeutic agents.

The lignan components identified in *Epimedium* comprise twelve structural types, including monoepoxy lignans, propyl neolignans, rhamnosides, podophyllotoxin A, podophyllotoxin C, rhododendrol, isopodophyllotoxin, and cycloeuphoral [34]. These compounds are predominantly distributed in the stems, leaves, and rhizomes of *Epimedium* and contribute to its pharmacological effects through synergistic interactions with flavonoids, alkaloids, and other constituents. Lignans are primarily lipophilic and possess complex molecular architectures, often conjugating with sugar moieties to form glycosides (e.g., rhamnosides), thereby influencing their bioavailability.

### 2.5. Phytosterols and Terpenoids

The phytosterols found in *Epimedium* are primarily composed of β-sitosterol, with additional compounds including stigmasterol, campesterol, and daucosterol [34]. These phytosterols act synergistically with other bioactive constituents, such as icariin, to produce various pharmacological effects. Terpenoids, natural organic compounds composed of isoprene units, occur at relatively low concentrations in *Epimedium*. Oleanolic acid, a pentacyclic triterpenoid compound extracted from *Epimedium wushanense* T. S. Ying, commonly exists in both free and glycoside forms with concentrations generally ranging from 0.2% to 2% [34].

**Table 1:** The bioactive components that have been identified in *Epimedium* (Note: ^a^ Rep. Comp., representative compounds; ^b^ Mol. For., molecular formula; ^c^ Mol. Wt., molecular weight; ^d^ Ref., references).

Species	^a^ Rep. Comp.	^b^ Mol. For.	^c^ Mol. Wt.	Plant	Part	Abundance	Pharmacokinetic Exposure	^d^ Ref.
Flavonoids	Icariin	C_33_H_40_O_15_	676.7	*Epimedium brevicornum* Maxim., *Epimedium sagittatum* (Siebold & Zucc.) Maxim., *Epimedium pubescens* Maxim., *Epimedium koreanum* Nakai, and *Epimedium wushanense* T. S. Ying	Whole	1155.98–4252.89 μg/g across five-species means; 1.28–11.21 mg/g across East-Asian species/origins; 8.50–39.9 mg/g in 419 *Epimedium brevicornu* Maxim. samples.	*Epimedium* total flavonoid capsule (ETFC) (derived from the extraction of *Epimedium brevicornu* Maxim.) rat study: among highest plasma exposure: T_max_ 0.22 ± 0.02 h, C_max_ 18.22 ± 7.16 ng/mL, AUC_0–∞_ 62.32 ± 21.59 ng·h/mL; brain AUC_0–t_ 35.36 ± 5.56 ng·h/g.	[38,42,44,77,78,79]
	Icaritin	C_21_H_20_O_6_	368.4	*Epimedium brevicornum* Maxim., *Epimedium sagittatum* (Siebold & Zucc.) Maxim., *Epimedium pubescens* Maxim., *Epimedium koreanum* Nakai, and *Epimedium wushanense* T. S. Ying	Leaves	17.45–73.76 μg/g across five-species means; a 34-accession survey detected only 0.01–0.08 mg/g in three accessions.	ETFC rat study: T_max_ 8.67 ± 1.12 h, C_max_ 2.24 ± 0.43 ng/mL, AUC_0–∞_ 24.67 ± 5.34 ng·h/mL.	[47,77,78,80]
	Icariside I	C_27_H_30_O_11_	530.5	*Epimedium brevicornum* Maxim., *Epimedium sagittatum* (Siebold & Zucc.) Maxim., *Epimedium pubescens* Maxim., *Epimedium koreanum* Nakai, and *Epimedium wushanense* T. S. Ying	Aerial	4.76–36.24 μg/g across five-species means	ETFC rat study: plasma T_max_ 0.29 ± 0.04 h, C_max_ 0.25 ± 0.05 ng/mL, AUC_0–∞_ 0.27 ± 0.02 ng·h/mL; brain AUC_0–t_ 0.41 ± 0.15 ng·h/g.	[54,60,77,78,80]
	Icariside II	C_27_H_30_O_10_	514.5	*Epimedium brevicornum* Maxim., *Epimedium sagittatum* (Siebold & Zucc.) Maxim., *Epimedium pubescens* Maxim., *Epimedium koreanum* Nakai, and *Epimedium wushanense* T. S. Ying	Leaves	64.84–215.37 μg/g across five-species means; 0.19–3.23 mg/g across selected East-Asian materials; up to 4.48 mg/g in a 34-accession survey.	ETFC rat study: high plasma exposure and brain AUC_0–t_ 33.39 ± 6.32 ng·h/g, comparable with icariin despite lower extract content. Rat absolute oral bioavailability reported as 4.1% versus 0.1% for icariin. Human serum: principal circulating metabolite after standardized extract.	[42,54,77,78,79,80,81]
	Epimedin A	C_39_H_50_O_19_	822.8	*Epimedium brevicornum* Maxim., *Epimedium sagittatum* (Siebold & Zucc.) Maxim., *Epimedium pubescens* Maxim., *Epimedium koreanum* Nakai, and *Epimedium wushanense* T. S. Ying	Whole	1254.84–2138.93 μg/g across five-species means; 2.31–8.42 mg/g in 419 *Epimedium brevicornu* Maxim.	ETFC rat study: rapid systemic absorption; brain AUC_0–t_ 5.02 ± 0.98 ng·h/g. Oral *Epimedium* extract studies also report measurable plasma exposure.	[38,77,78,82,83]
	Epimedin B	C_38_H_48_O_19_	808.8	*Epimedium brevicornum* Maxim., *Epimedium sagittatum* (Siebold & Zucc.) Maxim., *Epimedium pubescens* Maxim., *Epimedium koreanum* Nakai, and *Epimedium wushanense* T. S. Ying	Whole	1111.82–2370.28 μg/g across five-species means; 6.67–55.7 mg/g in 419 *Epimedium brevicornu* Maxim. samples.	ETFC rat study: measurable plasma exposure; brain C_max_ 10.68 ± 3.62 ng/g and AUC_0–t_ 18.35 ± 2.92 ng·h/g.	[38,77,78,83,84]
	Epimedin C	C_38_H_50_O_19_	822.8	*Epimedium brevicornum* Maxim., *Epimedium sagittatum* (Siebold & Zucc.) Maxim., *Epimedium pubescens* Maxim., *Epimedium koreanum* Nakai, and *Epimedium wushanense* T. S. Ying	Whole	13.02–71.62 μg/g across five-species means; trace/low in a 34-accession survey.	ETFC rat study: among plasma exposure: T_max_ 1.80 ± 1.04 h, C_max_ ng/mL, AUC_0–∞_ 24.68 ± 5.50 ng·h/mL; brain C_max_ 17.49 ± 6.04 ng/g and AUC_0–t_ 28.53 ± 4.51 ng·h/g.	[38,42,55,77,78,83]
	Hyperoside	C_21_H_20_O_12_	464.4	*Epimedium brevicornum* Maxim., *Epimedium sagittatum* (Siebold & Zucc.) Maxim., *Epimedium pubescens* Maxim., *Epimedium koreanum* Nakai, and *Epimedium wushanense* T. S. Ying	Aerial	98.85–201.18 μg/g across five-species means; no significant difference among the five species in one survey (*p* = 0.234).	/	[60,77,80]
	Astragalin	C_21_H_20_O_11_	448.4	*Epimedium brevicornum* Maxim., *Epimedium sagittatum* (Siebold & Zucc.) Maxim., *Epimedium pubescens* Maxim., *Epimedium koreanum* Nakai, and *Epimedium wushanense* T. S. Ying	leaves	32.88–180.32 μg/g across five-species means; trace/low in a 34-accession survey.	/	[62,77]
	Quercitrin	C_15_H_10_O_7_	302	*Epimedium brevicornum* Maxim., *Epimedium sagittatum* (Siebold & Zucc.) Maxim., *Epimedium pubescens* Maxim., *Epimedium koreanum* Nakai, and *Epimedium wushanense* T. S. Ying	Whole	About 0.0035 ± 0.0009 g/100 g ethanol extract (about 35 ± 9 μg/g extract) in the manuscript-cited 37-specimen survey; dry-herb equivalent not reported.	/	[34,77,80]
	Kaempferol	C_27_H_30_O_14_	578.6	*Epimedium sagittatum* Maxim.	Whole	/	/	[34,63]
	Ginkgetin	C_32_H_22_O_10_	566.5	*Epimedium koreanum* Nakai.	Aerial	/	/	[85,86]
	Bilobetin	C_31_H_20_O_10_	552.5	*Epimedium koreanum* Nakai.	Aerial	About 0.0062 ± 0.0019 g/100 g ethanol extract (about 62 ± 19 μg/g extract) in the manuscript-cited 37-specimen survey; dry-herb equivalent not reported.	/	[31,86]
	Apigenin	C_15_H_10_O_5_	270.2	*Epimedium brevicornum* Maxim.	Whole	5.54–30.99 μg/g across five-species means.	/	[63,87]
	Luteolin	C_15_H_10_O_6_	286.2	*Epimedium brevicornum* Maxim., *Epimedium sagittatum* (Siebold & Zucc.) Maxim., *Epimedium pubescens* Maxim., *Epimedium koreanum* Nakai, and *Epimedium wushanense* T. S. Ying.	Whole	/	/	[34,77]
	Daidzein	C_15_H_10_O_4_	254.2	*Epimedium sutchuenense* Franch.	Leaves	/	/	[34]
Coumarin	5,7,4,-Trimethoxy-4-phenylcoumarin	C_18_H_16_O_5_	312.3	*Epimedium brevicornum* Maxim.	Leaves	706.85–1369.82 μg/g across five-species means (sample range 470.45–2165.07 μg/g); a broader 80-sample study reported 0.003–2.603% in leaves across species/parts/times.	/	[34,88]
Alkaloid	Magnoflorine	C_20_H_24_NO_4_	342.4	*Epimedium koreanum* Maxim.	Whole	/	Oral *Epimedium* extract in rats: dual plasma peaks, C_max_ about 411 and 276 ng/mL, t_1/2_ 17.77 h, AUC_0–∞_ 4003 ng·h/mL.	[34,77,83,89]
Phytosterol	β-sitosterol	C_29_H_50_O	414.7	*Epimedium koreanum* Maxim.	Whole	/	/	[34,90]
	Daucosterol	C_35_H_60_O_6_	576.9	*Epimedium koreanum* Maxim.	Whole	/	/	[34]
Terpenoids	Oleanolic acid	C_30_H_48_O_3_	456.7	*Epimedium wushanense* T. S. Ying	Root	249.06–664.78 μg/g across five-species means.	/	[34]
Phenylpropanoid	Chlorogenic acid	C_16_H_18_O_9_	354.3	*Epimedium sagittatum* Maxim. and *Epimedium koreanum* Maxim.	Whole	/	Oral *Epimedium* extract in rats: C_max_ about 540 ng/mL, t_1/2_ 0.45 h, AUC_0–∞_ about 886 ng·h/mL.	[34,77,83]
	Protocatechuic acid	C_7_H_6_O_4_	154.1	*Epimedium koreanum* Maxim.	Aerial	2.98–18.73 μg/g across five-species means.	/	[34]
	Caffeic acid	C_9_H_8_O_4_	180.2	*Epimedium koreanum* Maxim.	Aerial	/	/	[34,77]
	Stachydrine	C_7_H_14_NO_2_	144.2	*Epimedium acuminatum* Franch.	Aerial	/	/	[26,34]

## 3. Bone-Related Biological Functions of the Active Constituents

### 3.1. Osteogenic Differentiation Regulation

Osteogenic differentiation regulation serves as a central driving force in the process of bone regeneration, facilitating functional bone remodeling through multi-dimensional coordination of cellular behaviors and interactions with the surrounding microenvironment. This regulatory mechanism is primarily reflected in three key aspects: Firstly, by activating specific transcriptional programs, it directs stem cells to undergo lineage-specific differentiation into osteogenic cells, thereby ensuring a continuous supply of bone-forming cells [91,92]. Secondly, it governs the dynamic synthesis and remodeling of the extracellular matrix, leading to the formation of a mineralized scaffold that supports vascularization and cell migration, while the mechanical properties of the matrix provide feedback to reinforce the differentiation process [91,92]. Finally, it integrates signals from the microenvironment and coordinates the temporal interplay between inflammation resolution and tissue reconstruction, establishing conditions favorable for regeneration [91,92]. This multi-level regulatory network not only sustains homeostasis during bone regeneration but also offers critical therapeutic targets for clinical intervention. Enhancing the efficiency of this regulatory process through physical, chemical, or biological strategies can significantly improve the outcomes of repairing complex bone defects [91,92].

#### 3.1.1. Flavonoids

Stem cells, particularly bone marrow-derived MSCs (BMSCs), have the capability to transform into osteoblasts and chondrocytes under proper inductive conditions, making them a significant biological resource for the repair of bone defects. Furthermore, stem cells can enhance cellular proliferation and extracellular matrix production by secreting bioactive chemicals, such as bone morphogenetic proteins (BMPs), OCN, osteopontin (OPN), and Col I [93,94]. Flavonoids derived from *Epimedium* have demonstrated the ability to augment cell proliferation, motility, and differentiation in the osteogenic differentiation of stem cells. Jiao et al. established that icariin promotes the migration of BMSCs both in vivo and in vitro through the activation of the MAPK signaling pathway [95]. Tang et al. reported that in a rat model of osteoporosis, the combination of icariin and stem cells significantly increases bone mass and improves bone microarchitecture [96]. Furthermore, icariin can activate the Ras homolog family member A (RhoA)-transcriptional coactivator with PDZ-binding motif (TAZ) signaling pathway to promote the proliferation and osteogenic differentiation of rat adipose-derived stem cells (ASCs), with 0.1 μM identified as the optimal concentration [18]. Under conditions of *Porphyromonas gingivalis* infection, icariin can downregulate the erythropoietin-producing hepatocellular A2 (EphA2)-RhoA signaling pathway, thereby enhancing osteogenic differentiation and bone regeneration, with 10 μM icariin showing the most pronounced effect [97]. Research has demonstrated that icariside II can enhance the osteogenic differentiation of BMSCs, with this effect being at least partially mediated through the modulation of MAPK/extracellular signal-regulated kinase (ERK) signaling pathway activation [98]. Additionally, apigenin has been shown to promote the osteogenic differentiation of BMSCs via the c-Jun N-terminal kinase (JNK) and p38 MAPK signaling pathways [99]. Furthermore, icaritin can significantly increase the expression levels of osteogenesis-related mRNAs in human BMSCs, with notable effects observed at concentrations ranging from 10–7 to 10–5 M [100]. It has been reported that hyperoside promotes the proliferation and osteogenic differentiation of BMSCs by activating the NF-κB signaling pathway, leading to improvements in experimental periodontitis in rats [101]. In osteoporotic models, kaempferol has been shown to enhance the osteogenic differentiation of MSCs by down-regulating miR-10a-3p, up-regulating C-X-C motif chemokine ligand 12 (CXCL12), or inhibiting caveolin-1 expression [102,103]. Quercitrin can increase alkaline phosphatase (ALP) activity and mineralization in human BMSCs, thereby promoting their differentiation toward the osteoblastic lineage [104].

It is widely recognized that the differentiation of BMSCs into either osteoblasts or adipocytes is determined by the coordinated regulation of multiple signaling pathways [105,106]. However, an increase in bone marrow adiposity has been associated with a higher risk of fractures [107]. Although icariin lacks direct estrogenic activity, it has been reported to exhibit properties similar to those of selective estrogen receptor (ESR) modulators [108]. Consequently, icariin can indirectly promote osteogenic differentiation while suppressing adipogenic differentiation in rat BMSCs through modulation of the ESR alpha (ESRα) signaling pathway. Furthermore, icariin has been shown to restore osteogenic differentiation and enhance bone formation in bone marrow stromal cells within an estrogen-deficiency-induced osteoporotic rat model [109]. Also, icaritin not only enhances the proliferation and osteogenic differentiation of BMSCs, but also suppresses their adipogenic differentiation, thereby promoting periosteal bone formation [14,52,100,110,111,112,113,114,115,116,117].

Osteoblasts are specialized bone-forming cells within bone tissue, typically derived from BMSCs. Although osteoblasts are not terminally differentiated cells, they undergo further differentiation into osteocytes upon appropriate induction. As key effector cells in bone formation, osteoblasts play a central role in bone remodeling and repair by synthesizing osteoid, initiating mineralization, and participating in cellular signal transduction. Dysregulation of osteoblast function can contribute to pathological conditions such as osteoporosis and osteosclerosis. It has been reported that icariin can rapidly induce phosphorylation of ESRα at the serine 118 and serine 167 residues in osteoblasts via the MAPK/ERK and phosphoinositide 3-kinase (PI3K)/protein kinase B (AKT) signaling pathways, respectively [108]. Moreover, icariin can regulate the anabolic metabolism of osteoblasts by up-regulating the expression of BMP-2, BMP-4, and drosophila mothers against decapentaplegic homolog 4 (Smad4) [118,119,120]. Studies have demonstrated that icariin may enhance fracture healing in both young and aged rats through activation of the BMP-2/Smad5/Runx2 pathway [121]. Additionally, icariin has been shown to prevent glucocorticoid-induced osteoporosis by increasing the expression of differentiated embryonic chondrocyte expressed gene 1 [122]. Wu et al. reported that icariin can promote bone formation in rats with chronic high-dose alcohol-induced osteoporosis, inhibit bone loss, and effectively restore bone architecture and mechanical strength [123]. Primary cilia are evolutionarily conserved structures present in bone cells, including osteoblasts and chondrocytes, and play essential roles in osteogenesis and cartilage development [124,125,126]. Accumulating evidence suggests that primary cilia are closely associated with the onset and progression of diabetes mellitus [127,128]. Liu et al. investigated diabetic-induced bone loss and found that icariin can reverse this condition by scavenging reactive oxygen species (ROS) and activating the Cilia/Gli2/OCN signaling pathway [129]. Moreover, icariin may play a potential role in the prevention and treatment of osteoporosis by upregulating the mRNA expression levels of OPN, BMPs, and Col I in OVX rats [130]. Notably, clinical studies have indicated that icariin can increase bone density and improve bone strength in postmenopausal women [131]. In addition to icariin, icaritin and icariside II has also been demonstrated to enhance the osteogenic differentiation of BMSCs, potentially via regulation of the MAPK/ERK signaling pathway [132]. Also, icaritin can induce the differentiation and maturation of MC3T3-E1 subclone 14 cells by activating MAPK signaling components, including ERK1/2 and p38 [133]. Lead exposure has been found to inhibit the differentiation of MC3T3-E1 subclone 14 cells [134]. This may be attributed to the inhibition of the canonical Wnt signaling pathway by lead through the regulation of key components, including Wnt3a, Dickkopf-1, p-glycogen synthase kinase 3β (pGSK-3β), and β-catenin [134]. As expected, icaritin treatment can partially restore Wnt/β-catenin signaling and counteract the inhibitory effects of lead on osteogenesis [134]. The focal adhesion kinase (FAK) signaling pathway is constitutively activated during the osteogenic differentiation of human MSCs, and its activity is further enhanced upon induction of osteoblastic differentiation [135,136]. However, FAK knockout has been shown to significantly attenuate the activation of the Wnt/β-catenin and MAPK signaling pathways and effectively inhibit BMP-9-induced bone formation in vivo [137]. Recent studies have shown that epimedin A and epimedin B can interact with FAK to modulate the signaling activity, offering therapeutic potential for osteoporosis [138]. Furthermore, RNA sequencing and KEGG enrichment analyses have revealed that epimedin B exerts its anti-osteoporotic effects by regulating the PI3K/AKT, MAPK, and peroxisome proliferator-activated receptor (PPAR) signaling pathways in mouse models [139]. Due to its structural similarity to icariin, epimedin C is hypothesized to possess comparable pharmacological properties [140]. It has been demonstrated that there are no significant differences in osteoblast function or osteogenic efficacy between epimedin C and icariin [140]. Both compounds exhibit high efficacy in lipopolysaccharide (LPS)-induced osteoblast models, where they can reverse LPS-mediated inhibition of osteoblast proliferation, mineralization, and BMP-2/Runx2 signaling [140]. Moreover, epimedin C has been shown to alleviate dexamethasone-induced suppression of bone formation in zebrafish larvae and MC3T3-E1 cells by activating the PI3K/AKT/Runx2 signaling pathway [141]. Epimedin C also protects osteoblasts from dexamethasone-induced damage by stabilizing the nuclear factor erythroid 2-related factor 2 (Nrf2)-RhoA promoter complex [142].

Hyperoside has been found to significantly activate the expression of acetylcholinesterase associated with proline-rich membrane anchor in osteoblasts [143]. Additionally, hyperoside can promote osteogenic differentiation under inflammatory conditions by simultaneously inhibiting NF-κB activation, β-catenin expression, and ROS production, exerting beneficial effects on human osteoblast-like cells (Saos-2 cells) [144,145]. Researchers have demonstrated that the extract of St. John’s wort, which primarily contains hyperoside, hypericin, and flavonoids, can significantly ameliorate forced swimming-induced stress injury in experimental animals and restore bone density to normal levels [146]. Astragalin has been reported to promote osteogenic differentiation of MC3T3-E1 cells via the BMP and MAPK signaling pathways and enhance bone formation in OVX-induced osteoporotic mice [147]. Studies have indicated that daidzein therapy improves osteoblast viability and speeds up the development of bone progenitor cells into fully developed osteoblasts. Daidzein enhances ALP activity, OCN production, and BMP-2 mRNA expression in primary osteoblast cells [148]. These effects are mediated through activation of the BMP signaling pathway, which upregulates the phosphorylation levels of Smad1/5/8 [149]. Daidzein also promotes the secretion of osteoprotegerin (OPG) in cultured normal porcine osteoblasts while reducing the membrane expression of RANKL [150]. In an OVX rat model of post-menopausal osteoporosis, Picherit et al. demonstrated that oral administration of daidzein can prevent the loss of trabecular and cortical bone or cortical bone alone [151]. Additional studies confirm that daidzein enhances osteoblast proliferation and differentiation via the BMP pathway, further supporting its role in promoting bone formation [149]. Daily intake of daidzein and tempeh has also been shown to improve calcium content, calcium transport, and bone metabolism in ovariectomized rats [152]. Dietary soy isoflavones, including genistein and daidzein, may influence calcium homeostasis by modulating bone calcium release and protecting common fracture-risk sites such as the hip and spine [153]. Apigenin has been shown to activate hydrogen peroxide-induced PI3K and AKT2 gene expression while reducing ERK2 expression, thereby enhancing cell survival and mitigating oxidative damage in osteoblasts [154]. Additionally, apigenin stimulates the generation of bone marrow-derived progenitor cells and increases the mRNA levels of osteogenic markers such as Runx2, Col I, and BMP-2 [155]. Luteolin significantly promotes osteoblast differentiation and mineralization through activation of multiple signaling pathways, including Wnt/β-catenin, ERK/low-density lipoprotein receptor-related protein 5/GSK-3β, and PI3K/AKT [156,157]. Network pharmacology study indicates that luteolin demonstrates multi-target effects, engaging pathways including PI3K-AKT, TNF, and estrogen signaling, thereby elucidating its molecular foundation for therapeutic efficacy in osteoporosis and glucocorticoid-driven avascular necrosis of the femoral head [158,159]. Moreover, luteolin enhances osteoblast activity by increasing the mRNA and protein expression levels of bone formation markers, including ALP, BMP-2, OPG, Osterix, and OCN, as well as the expression of β-catenin and cyclin D1 [160,161]. Nash et al. demonstrated that luteolin interacts with ESRs in mouse osteoblasts, resulting in increased ALP activity and collagen synthesis [157]. Yang et al. reported that luteolin inhibits IL-1β-induced expression of matrix metalloproteinases (MMP-9 and MMP-13) in mouse calvarial osteoblasts, potentially through suppression of the ERK pathway, thereby preventing excessive bone matrix degradation [162]. Notably, various concentrations of luteolin (0.01, 0.1, 1, 10, and 100 μmol/L) were found to enhance calcified nodule formation in human periodontal ligament cells. Among these, 1 μmol/L luteolin exhibited the most pronounced effect, promoting osteogenic differentiation via activation of the Wnt/β-catenin signaling pathway [161]. A polysaccharide purified from *Epimedium brevicornum* Maxim. has been identified with the following glycosidic linkages: →4)-α-D-Galp-(1→4)-α-D-Galp-(1→6)-β-D-Galp-(1→6)-β-D-Galp-(1→4)-α-D-Glcp-(1→4)-α-D-Glcp-(1→. In vitro assays demonstrate that this polysaccharide can enhance osteogenic differentiation in osteoblasts under high-glucose conditions. Moreover, the study suggests that polysaccharide treatment promotes osteogenesis in the context of diabetic osteoporosis, potentially through the regulation of the B-cell lymphoma/leukemia-2 (Bcl-2)/Bcl-2-associated X protein (Bax) pathway and the subsequent enhancement of osteoblastic activity [163]. Another polysaccharide isolated from *Epimedium brevicornum* Maxim., primarily composed of glucose, was used to pre-treat osteoblasts during glucocorticoid-induced osteoporosis. An in vitro study indicate that it exerts protective effects on osteoblasts by activating the PI3K/AKT/mammalian target of rapamycin (mTOR) and Wnt/β-catenin signaling pathways and suppressing mitochondrial apoptosis [164]. Quercitrin has been reported to alleviate osteoporosis by increasing ALP expression and inhibiting the JNK, ERK, and p38 MAPK signaling pathways [165]. Additionally, quercitrin protects osteoblasts from hydrogen peroxide (H_2_O_2_)-induced cytotoxicity and reverses oxidative stress-induced dysfunction [166]. Bilobetin has also been shown to enhance ALP activity, collagen synthesis, and calcium deposition, thereby promoting osteoblast mineralization [31]. Kaempferol activates the osteogenic signaling pathway via the Sox2/miR-124-3p/PI3K/AKT/mTOR axis, significantly increasing ALP activity and calcium deposition [167]. Network pharmacology studies further reveal that kaempferol participates in regulating the TNF signaling pathway, atherosclerosis-related signaling pathways, and the advanced glycation end products (AGEs)/receptor for AGEs (RAGE) signaling pathway through multiple targets such as AKT1 and MMP9, supporting its therapeutic potential in osteoporosis [168].

#### 3.1.2. Other Active Constituents

Kabashima et al. demonstrated that magnoflorine at a concentration of 25 μg/mL significantly enhances the viability, progression through the S phase, and mineralization capacity of MC3T3-E1 cells [169]. Oleanolic acid exerts anabolic effects on bone by activating 25-hydroxyvitamin D1-α-hydroxylase in osteoblasts. Furthermore, its bone-protective effects are associated with the induction of cytochrome P450 family 27 subfamily B member 1 activity and expression in bone tissue and within the osteoblast lineage [170]. Additionally, oleanolic acid promotes the osteogenic differentiation of MSCs by suppressing the Notch signaling pathway, thereby enhancing the expression of osteogenic markers such as ALP, Runx2, and Col I, which ultimately facilitates bone formation [171]. It also enhances osteoblast activity by upregulating OPG mRNA expression and modulating the phosphorylation of ERK, JNK, p38, and AKT [172]. Caffeic acid phenethyl ester demonstrates a positive regulatory effect on bone metabolism in C57BL/6J bone marrow-derived cells by promoting osteoblast differentiation and bone maturation. Moreover, low-concentration caffeic acid can enhance MSCs mineralization and upregulates the expression of osteogenic markers [173]. It also stimulates the production of BMPs and cathepsin B in osteogenic precursor cells [173]. Chlorogenic acid promotes the osteogenic differentiation of human adipose tissue-derived mesenchymal stem cells and human dental pulp stem cells (hDPSCs) via activation of the Wnt/Ca^2+^ signaling pathway and induction of calcium deposition [174]. Han et al. confirmed that chlorogenic acid protects osteoblasts from oxidative stress by modulating the PI3K/AKT signaling pathway and enhancing the expression of Nrf2/heme oxygenase-1 (HO-1) [175]. In addition, chlorogenic acid can mitigate dexamethasone-induced osteoblast apoptosis through activation of the Nrf2/HO-1 antioxidant signaling pathway [176]. Recent studies have also revealed that oral administration of chlorogenic acid promotes the proliferation of osteoprogenitor cells and the osteogenic differentiation of BMSCs via the SH2 domain-containing protein-tyrosine phosphatase-2/PI3K/AKT/cyclin D1 signaling cascade, thereby preventing osteoporosis in ovariectomized female rats [177]. At a dosage of 20 mg/kg, chlorogenic acid significantly improved femoral head and neck bone mineral density following ischemic injury [178]. This indicates its therapeutic potential for high-concentration treatment of hormone-induced femoral head necrosis. Notably, OVX was found to markedly reduce bone volume and mechanical integrity in rats, whereas chlorogenic acid administration effectively counteracted these adverse effects [179]. In vivo studies have further demonstrated that chlorogenic acid enhances osteoblast proliferation and differentiation, thereby promoting bone mineralization and improving bone mechanical strength [180,181]. These beneficial effects may be partially attributed to chlorogenic acid’s ability to amplify TNF-α-stimulated IL-6 synthesis in osteoblasts [182,183].

### 3.2. Inhibition of Osteoclast Differentiation/Bone Resorption

Osteoclast-mediated bone resorption is a crucial element of the bone remodeling cycle. Although maintaining osteoclast function is necessary for sustaining bone homeostasis, a certain degree of inhibition of osteoclast differentiation during bone repair may yield synergistic positive effects [184,185]. Firstly, it mitigates premature bone resorption, prolongs the collagen mineralization period, and protects the newly formed extracellular matrix [184,185]. Secondly, it disrupts the negative feedback regulation of bone remodeling, promotes mesenchymal stem cell differentiation toward the osteogenic pathway, and consequently enhances osteogenic dominance [184,185]. Thirdly, it prevents bone mass loss caused by the imbalance between resorption and formation, maintains the structural integrity of the regenerating bone tissue, and thereby improves the overall quality of bone repair [184,185].

#### 3.2.1. Flavonoids

To date, the most extensive and in-depth research has centered on the effects of three flavonoids derived from *Epimedium* (i.e., icariin, icaritin, and icariside) on osteoclastic bone resorption. In a study utilizing a rat OVX model, icariin was demonstrated to prevent bone loss and maintain bone strength post-OVX [186]. It dramatically enhances the expression of OPG while diminishing RANKL levels, hence increasing the OPG/RANKL ratio, suppressing osteoclast activity, and mitigating bone loss in OVX rats [187]. Recent studies have also indicated that icariin ameliorates osteoporosis in ovariectomized rats by modulating the Cullin3/Nrf2/HO-1 signaling pathway to suppress osteoclastogenesis [19]. Furthermore, icariin inhibits osteoclast differentiation and bone resorption by suppressing the synthesis of hypoxia-inducible factor-1α (HIF-1α) and prostaglandin E, which are regulated through the MAPK/NF-κB signaling pathway [187]. Additional research has demonstrated that icariin exerts inhibitory effects on titanium particle-induced inflammatory osteoclastogenesis, with an optimal concentration of 0.0148 M [188,189].

Tumor necrosis factor receptor-associated factor 6 (TRAF6) serves as a key adaptor protein in transducing signals from the RANKL/receptor activator of nuclear factor κB (RANK) axis, while nuclear factor of activated T cells c1 (NFATc1) functions as the master transcriptional regulator of osteoclast differentiation [15,190]. Icaritin suppresses osteoclast differentiation by downregulating TRAF6, which in turn inhibits the NF-κB, MAPK/activator protein 1 (AP-1), and ROS signaling pathways, leading to reduced expression and activity of NFATc1 [15]. Notably, studies have revealed that icaritin decreases mitochondrial mass and reduces the density of mitochondrial cristae. Additionally, icaritin attenuates mitochondrial membrane potential elevation and modulates mitochondrial fusion and fission dynamics, thereby inhibiting osteoclast differentiation and maturation [53]. Zhao et al. demonstrated that icaritin can inhibit lung cancer-induced osteoclastogenesis by downregulating the expression of IL-6 and TNF-α, as well as through modulation of the AMP-activated protein kinase (AMPK)/mTOR signaling pathway [191]. The parent ring structure of icaritin is structurally similar to that of phytoestrogens, featuring hydroxylation at the C-3 and C-7 positions, which confers estrogen-like activity [192,193,194]. Furthermore, preliminary experiments indicate that dietary treatment of icaritin could avoid osteoporosis linked to estrogen insufficiency [195]. Lin et al. reported that icaritin can alleviate motor dysfunction in ovariectomized Parkinson’s disease model mice through activating the ER/PI3K/AKT signaling pathway [196]. Also, Zhang et al. found that icaritin effectively mitigates OVX-induced bone loss and improves bone metabolism through the modulation of the toll-like receptor 4 (TLR4) and PI3K/AKT signaling pathways, thereby providing a potential therapeutic strategy for osteoporosis [195].

Regarding icariside I, it can directly inhibit osteoclast differentiation and bone resorption by suppressing the RANKL-induced TRAF6/MAPK/p38/NFATc1 signaling cascade [197]. Furthermore, icariside I can promote osteoblast differentiation and maturation in a dose-dependent manner by inhibiting adipogenesis and enhancing osteoblast energy metabolism [197]. Icariside I also significantly improves OVX-induced osteoporosis in mice by targeting the gut microbiota dysbiosis-Th1 cell-IFN-γ-osteoclast signaling axis [197]. Additionally, icariside I may inhibit osteoclast differentiation by promoting the production of short-chain fatty acids [198]. In comparison, icariside II exhibits higher inhibitory activity against osteoclast-mediated bone resorption [14]. Studies have also shown that icariside II exerts a stronger inhibitory effect on RANKL-induced osteoclast formation than icariin. This effect is likely due to the presence of a free hydroxyl group at the C-7 position, which enhances its inhibitory potential against osteoclast differentiation and bone resorption. The urokinase-type plasminogen activator receptor (uPAR) plays a crucial role in regulating osteoclast metabolism. Icariside II can prevent OVX-induced bone loss and attenuate RANKL-stimulated expression of NFATc1 and uPAR, thereby offering therapeutic benefits for osteoporosis [199]. Intriguingly, icariside II and icariin in *Epimedium* exhibit a synergistic inhibitory impact on the expansion of osteoclast precursor RAW264.7 cells [200]. A combination of 100 μM icariin and 100 μM icariside II achieves nearly a 90% inhibition rate on osteoclast formation [201]. And the bone resorption inhibition rates for these compounds were 50% and 80%, respectively [201].

Among other bioactive flavonoids in *Epimedium*, epimedin A has also been shown to inhibit osteoclast differentiation by suppressing the PI3K/AKT/NF-κB signaling axis through negative regulation of TRAF6 expression [202]. Hyperoside exerts anti-osteoporotic effects in OVX mice by inhibiting the TRAF6-mediated RANKL/RANK/NF-κB signaling pathway, increasing the OPG/RANKL ratio, and modulating the ERα/ITGβ3 signaling pathway [203]. Hyperoside also improves bone architecture in OVX mice by enhancing miR-19a-5p-mediated downregulation of IL-17A, thereby exerting a protective effect against post-menopausal osteoporosis [204]. By inhibiting the MAPK and ROS pathways, astragalin disrupts osteoclast differentiation [205]. Consequently, it decreases inflammatory osteolysis by downregulating critical osteoclast markers such as proto-oncogene c-Fos (c-Fos), NFATc1, cathepsin K (CTSK), and tartrate-resistant acid phosphatase (TRAP) [205]. Studies have confirmed that ginkgetin inhibits bone loss in OVX mice, potentially through suppression of the NF-κB/inhibitory subunit of NF kappa B alpha (IκBα) signaling pathway, which inhibits osteoclast formation and maturation [85]. Ikarisoside A inhibits osteoclast differentiation of RAW264.7 cells via the JNK and NF-κB signaling pathways [206]. Daidzein plays a significant role in bone metabolism by effectively increasing bone density in postmenopausal women and preventing bone loss, potentially through enhancing the expression of ESRα, ESRβ, and steroid receptor coactivator-1 in the estrogen response element pathway, thereby promoting its activation [149,207,208]. Administration of daidzein to OVX mice suppress osteoclastogenesis by significantly reducing ROS and TNF-α levels produced by activated T cells [209]. Furthermore, neonatal exposure to daidzein, genistein, or their combination has been shown to positively influence skeletal development in female mice, without exerting significant effects in male mice [210]. In mouse bone marrow-derived macrophages, luteolin inhibits RANKL-induced osteoclast differentiation and downregulates the expression of osteoclast-related genes, including NFATc1, c-Src tyrosine kinase, dendritic cell-specific transmembrane protein, MMP-9, CTSK, and TRAP [211]. In mature osteoclasts stimulated with RANKL and macrophage colony-stimulating factor (M-CSF), luteolin inhibits bone resorption in a dose-dependent manner [211]. In RAW264.7 cells, luteolin suppresses the formation of RANKL-induced mature TRAP-positive osteoclasts by inhibiting the p38 MAPK and activating transcription factor 2 signaling downstream of NFATc1, thereby reducing bone resorption [212]. Coumarin inhibits osteoclastogenesis-related gene expression by modulating signaling pathways such as NF-κB/MAPK/AKT/Ca^2+^ [213]. Coumarin improves diabetic bone metabolism by regulating the RANK-RANKL signaling axis in RAW264.7 cells [214]. Additionally, the activation of Nrf2 by apigenin can mitigate oxidative stress in BMSCs and inhibit osteoclast-mediated bone resorption, contributing to its anti-osteoporotic effects [87]. Kaempferol suppresses IL-1β-stimulated, RANKL-mediated osteoclastogenesis by downregulating MAPK, c-Fos, and NFATc1 signaling [215].

#### 3.2.2. Other Active Constituents

In vitro, magnoflorine can inhibit RANKL-stimulated inflammatory responses and osteoclastogenesis by suppressing the phosphorylation of transforming growth factor-β (TGF-β)-activated kinase and the activation of downstream MAPK and NF-κB signaling pathways [216]. As expected, it alleviates titanium particle-induced inflammatory osteolysis in vivo [216]. Boiogito is a herbal formulation rich in magnoflorine used to treat knee osteoarthritis through the inhibition of osteoclast differentiation and inflammation [217]. Zhang et al. and Jin et al. reported that a specific concentration of β-sitosterol may reduce osteoclast numbers [218,219]. Studies have also indicated that β-sitosterol inhibits osteoclast migration, formation, and function by downregulating components of the cyclic adenosine monophosphate (cAMP) and NF-κB signaling pathways, thereby reducing osteoclast-mediated bone resorption and preventing OVX-induced bone loss [220]. Additionally, β-sitosterol protects Wistar rats against glucocorticoid-induced osteoporosis by modulating the RANKL/OPG and Runx2 signaling pathways [221]. Moreover, β-sitosterol suppresses IL-6 secretion and RANKL-induced TRAP activity [222]. Oleanolic acid inhibits RANKL-induced osteoclast formation via the ERα/miR-503/RANK signaling pathway and remains effective even at the late stages of osteoclastogenesis, preventing bone loss in bone-protective protein knockout mice [223,224]. Protocatechuic acid inhibits osteoclast differentiation by regulating the NF-κB and Nrf2 signaling pathways [225]. It has further been demonstrated to suppress osteoclast differentiation by modulating oxidative stress and inflammation and induces apoptosis in mature osteoclasts [225]. Jang et al. found that protocatechuic acid blocks the RANK signaling pathway by downregulating TRAF6 and NFATc1 expression, reduces serum RANKL levels, increases OPG levels, and attenuates the expression of CTSK and calcitonin receptor, thereby inhibiting bone loss in OVX mice [226]. Additionally, protocatechuic acid can inhibit osteoclast formation by suppressing the JNK/c-Fos/NFATc1 signaling pathway and thus prevent inflammatory bone loss in mice [227]. Caffeic acid inhibits RANKL-induced osteoclast formation and differentiation by suppressing the expression of osteoclast markers such as TRAP, CTSK, and MMP-9 [173]. Moreover, caffeic acid can regulate apoptosis-related bone loss in osteoclasts [173]. It has also been reported that caffeic acid inhibits the expression of NFATc1 and CTSK genes to suppress osteoclastogenesis [228]. Chlorogenic acid has been shown to inhibit RANKL-mediated osteoclast formation [229]. Furthermore, chlorogenic acid can also effectively prevent the formation and destruction of osteoclasts in myeloma cells induced by RANKL [179,230,231]. Osteoclasts require the capacity to produce protons to mediate bone resorption. Notably, the ruffled borders of osteoclasts contain vacuolar H-adenosine triphosphatase (V-ATPase), which hydrolyzes ATP to generate protons and transports them into the extracellular space. V-ATPase interacts with TRAF6, which is recruited by RANK, thereby facilitating its activation [232]. Lee et al. demonstrated that chlorogenic acid partially prevents the interaction between V-ATPase and TRAF6, thereby attenuating osteoclast differentiation and reducing bone resorption [233]. Endocytosis, secretion, and transport processes occurring at the ruffled borders of osteoclasts may be associated with cellular autophagy [234]. The formation of ruffled borders requires the coordinated action of several autophagy-related proteins and microtubule-associated protein light chain 3 to form actin rings, release CTSK, and promote osteoclastic bone resorption [235,236]. Chlorogenic acid may participate in this process by modulating autophagy to inhibit osteoclast activity [229].

### 3.3. Inflammation Regulation

In the course of biological evolution, the inflammatory response and tissue regeneration have co-evolved into a dialectical unity that is essential for effective tissue repair. As a tightly regulated biological process, successful bone regeneration fundamentally relies on the precise transition of the inflammatory phase from a destructive to a constructive state [237]. During the early stages, inflammation functions in a manner analogous to a scalpel, removing necrotic tissue and establishing a clean microenvironment conducive to regeneration [237]. Subsequently, as inflammation resolves in a timely fashion, it operates akin to an architect, orchestrating the migration and differentiation of reparative cells in a spatially and temporally coordinated manner [237]. This spatiotemporal regulation reflects the intrinsic self-organizing capacity of biological systems.

Achieving optimal bone regeneration necessitates the maintenance of inflammation within a so-called “golden range”: excessive inflammation may result in chronic inflammation, tissue damage, and fibrosis, whereas insufficient inflammation is inadequate to initiate the essential repair mechanisms [238]. Studies have demonstrated that successful bone healing is typically characterized by an inflammatory kinetic profile that follows a “rise first and then fall” pattern, reflecting the non-linear, self-regulating nature of biological systems through negative feedback mechanisms [238]. Clinically, the majority of refractory nonunion cases are associated with dysregulated inflammatory responses, further highlighting the critical importance of preserving this delicate balance.

#### 3.3.1. Flavonoids

A close interaction occurs between immune regulation and bone metabolism, creating an “anti-inflammatory-osteogenic” collaborative network that is crucial for the maintenance of bone equilibrium. Macrophages display immunomodulatory roles that facilitate proper bone repair [239,240]. A relevant study indicated that a suitable dose of icariin not only reinstates the reduced osteogenic potential of aged BMSCs but also mitigates the inflammatory senescence of aged macrophages through the activation of autophagy [241]. Shao et al. demonstrated that icariin prevents inflammatory responses and bone resorption induced by titanium particles in a murine calvarial model [242]. And Zuo et al. found that icariin diminishes excessive ROS production in chondrocytes by activating the Nrf2/antioxidant response element (ARE) signaling pathway and suppresses IL-1β-induced matrix breakdown in an osteoarthritis model [243]. Additional research confirmed that icariin can mitigate osteoarthritis by inhibiting the NOD-like receptor family pyrin domain-containing 3 (NLRP3) inflammasome-mediated caspase-1 signaling cascade [244]. Also, icariin exerts immunomodulatory effects by inhibiting the NF-κB/NLRP3 pathway and reducing the levels of pro-inflammatory cytokines such as IL-1β, IL-6, and TNF-α in a model of degenerative intervertebral disc disease [23,24]. Icariin inhibits cartilage and bone degradation in a collagen-induced arthritis murine model via suppressing CTSK activity [245]. Moreover, icariin activates miR-186 and miR-206 to improve dexamethasone-induced bone metabolic abnormalities [246,247]. One study revealed that icariin restores LPS-induced bone loss by downregulating miR-34c expression and inhibiting the JNK, p38, and NF-κB signaling pathways [22]. Furthermore, icariin significantly promotes the differentiation of MSCs into chondrocytes, enhances chondrocyte proliferation and the expression of related genes, reduces MMPs levels, and increases extracellular matrix (ECM) secretion, thereby facilitating cartilage repair [248,249]. Zhou et al. found that epimedin C ameliorates prednisolone-induced bone tissue damage [250]. The mechanism likely involves the activation of the PPAR and AMPK signaling pathways [250]. Apigenin can modulate inflammatory cytokines and the TGF-β and BMP signaling pathways to promote dentin formation and inflammation control [251]. PI3K regulates key cancer-related processes, including cell survival, proliferation, migration, protein synthesis, glucose homeostasis, and genomic instability [252,253]. Activation of PI3K is often driven by mutations in the p110α subunit [254]. Research has identified p110α as a novel therapeutic target for osteoarthritis, with its mechanism involving the inhibition of MMP13 overexpression via the PI3K/AKT/mTOR signaling pathway [255]. Specifically, p110α reduces cartilage ECM degradation and eases osteoarthritis via activating the PI3K/AKT/mTOR pathway [256]. Quercitrin activates the p110α/AKT/mTOR signaling pathway by targeting p110α, demonstrating promise in halting the progression of osteoarthritis by hindering the degradation of cartilage extracellular matrix and encouraging the proliferation of chondrocytes [256]. Furthermore, quercitrin demonstrates therapeutic potential against osteoarthritis through in vivo suppression of MMP13 and enhancement of type II collagen (Col II) deposition [256]. A study of Jin et al. also found that hyperoside reduces the production of pro-inflammatory cytokines and inhibits the NF-κB signaling pathway, thereby exerting anti-inflammatory effects and preventing joint destruction [257]. Moreover, this compound can mitigate TNF-α-induced inflammation, ECM degradation, and endoplasmic reticulum stress-mediated apoptosis in vitro through the sirtuin 1 (SIRT1)/NF-κB and Nrf2/ARE signaling pathways [258]. Local administration of hyperoside can partially restore the expression of forkhead box protein O1 and Runx2, thereby reducing alveolar bone resorption and periodontal ligament damage [144]. Astragalin suppresses MMPs expression by inhibiting the JNK/p38/AP-1 signaling pathway, significantly alleviating synovial inflammation and joint destruction in collagen-induced arthritis (CIA) mice [259]. Luteolin mitigates gingival fibroblast inflammation by downregulating the MAPK pathway in periodontitis and enhances the osteogenic differentiation of periodontal ligament stem cells through the Wnt/β-catenin signaling pathway, consequently decreasing the levels of inflammatory cytokines (TNF-α and IL-6) in a periodontitis model [20]. Kaempferol, when combined with flavonoids such as quercetin and apigenin, can synergistically enhance anti-inflammatory effects. For example, in an osteoarthritis model combined with stem cell therapy, kaempferol significantly reduces cartilage degradation markers (MMP-3 and MMP-13) and promotes collagen synthesis [260]. Additionally, kaempferol suppresses IL-1β-induced inflammation in rat osteoarthritis chondrocytes by inhibiting the NF-κB signaling pathway [261]. It also modulates the X chromosome inactivation-specific transcription factor/miR-130a/signal transducer and activator of transcription 3 (STAT3) axis in human chondrocyte C28/12 cells, effectively attenuating inflammation and ECM degradation [262].

#### 3.3.2. Other Compounds

As a potential inhibitor of the NF-κB signaling pathway, magnoflorine can exert significant anti-inflammatory effects through the MAPK signaling pathway [263]. Additionally, by partially blocking the PI3K/AKT/NF-κB axis signaling route and activating the kelch-like ECH-associated protein 1/Nrf2/HO-1 signaling pathway in vitro and in vivo, the compound can lower the inflammatory response in rheumatoid arthritis [264]. By triggering the signal transducer and activator of transcription 1 and NF-κB signaling pathways, β-sitosterol reduces pro-inflammatory cytokines and increases IL-10 in LPS-activated J774A.1 macrophages, thereby exerting anti-inflammatory effects [265,266,267]. Zhang et al. confirmed that protocatechuic acid suppresses the MAPK, AKT, and NF-κB signaling pathways, which decreases osteoclastogenesis and reduces osteoarthritis caused by anterior cruciate ligament transection [268]. Huang et al. demonstrated that chlorogenic acid not only significantly inhibits the expression of cyclooxygenase-2 and inducible nitric oxide synthase (iNOS) genes in macrophages but also reduces the secretion of inflammatory cytokines, including IL-1β, TNF-α, and IL-6 [269].

### 3.4. Antioxidation Effects

From an evolutionary perspective, the antioxidant mechanism represents the primordial strategy employed by living organisms to counteract the increase in entropy [270]. Re-establishing redox homeostasis during bone repair essentially involves modulating the “metabolic breathing rhythm” to reawaken the spatiotemporal memory embedded in the developmental program [270]. This therapeutic approach extends beyond symptomatic relief and directly addresses the core driving force underlying tissue regeneration—restoring to the injured site the self-organizing capacity observed during embryonic development [270]. In the oxidative stress environment induced by trauma, the antioxidant system functions as a biological firewall, preserving genomic stability and mitochondrial functional integrity in osteoblasts [271]. Such protection not only sustains the proliferative potential of osteoprogenitor cells but also ensures fidelity in epigenetic regulation during cellular differentiation, thereby supplying high-quality cellular components for bone regeneration [271]. The polarization dynamics of macrophages are critically influenced by redox equilibrium [272,273]. Appropriate antioxidant intervention can establish a conducive immune microenvironment that not only mitigates secondary tissue damage caused by excessive inflammatory responses but also facilitates coordinated vascular regeneration during the repair phase, thereby enabling precise transition between the “inflammation-repair” biphasic process [272,273]. ROS can act as sources of cellular damage as well as physiological signals [274,275,276,277,278,279,280,281]. When produced transiently at controlled levels within defined subcellular compartments, ROS function as redox messengers [274,275,282]. Low levels of Nox4-derived ROS mediate BMP-2-induced osteoblast differentiation and matrix mineralization in mouse pre-osteoblasts [283]. In addition, Endogenous ROS generated during osteogenic induction contribute to osteoblast differentiation and matrix mineralization in MC3T3-E1 cells [284]. Moderate levels of ROS also contribute to antimicrobial defense, angiogenesis, and adaptive stress responses [285,286,287]. Persistent or excessive ROS, however, can inhibit osteoblast proliferation and differentiation and increase osteoclast formation and bone resorption [288,289]. Complete or indiscriminate ROS depletion may also interfere with normal redox signaling and ROS-dependent host defense [290]. Antioxidant approaches to bone repair should therefore restore redox homeostasis at the appropriate site and time rather than eliminate ROS completely [291]. Their effects depend on the dose and timing of treatment, the cellular source of ROS, and conditions within the local microenvironment [291].

#### 3.4.1. Flavonoids

ROS can participate in the process of ferroptosis, an iron-dependent form of programmed cell death [292]. Ferroptosis, however, has been shown to induce bone loss in mice and contribute to osteoporosis development [293]. The role of Nrf2 in osteoblast differentiation may be related to intracellular ROS levels. Nrf2 deficiency in stromal cells leads to increased ROS accumulation, which induces oxidative stress and ultimately inhibits osteoblast differentiation [176,294]. It is reported that icariin may regulate osteoblast ferroptosis by activating the Nrf2/HO-1 signaling pathway, thereby promoting the healing of osteoporotic fractures [295]. Recent evidence indicates that icariin can reduce iron deposition in the bone marrow of osteoporotic mice and inhibit iron overload-induced osteoblast apoptosis, thereby increasing bone mass [296]. Lipid peroxidation has been identified as a key mechanism in iron overload-induced osteoporosis. Interestingly, the anti-osteoporotic effects of icaritin and its glycosides are closely related to their ability to inhibit lipid peroxidation. Yan et al. found in their study on the inhibition of lipid peroxidation by icaritin and its glycosides in zebrafish that the inhibition of lipid peroxidation by icaritin and its glycosides is achieved by suppressing oxidative stress and enhancing the activity of glutathione peroxidase 4 [297]. Intracellular ROS is involved in the differentiation process of osteoclasts, and nicotinamide adenine dinucleotide phosphate oxidase (NOX) is a key source of ROS [298]. Blocking NOX can improve ROS generation and the activation of downstream MAPK (JNK, p38, and ERK) and NF-κB, thereby inhibiting osteoclast formation [276,298]. A study has confirmed that icariin inhibits the generation of ROS by reducing the expression of NOX1 and NOX4, thereby inhibiting osteoclastogenesis in RANKL-induced RAW264.7 cells [299]. Oxidative stress can inhibit osteoblast differentiation, and reducing ROS levels can reverse the osteoclast differentiation induced by RANKL stimulation [276,300]. Hyperoside inhibits apoptosis and enhances osteoblast viability by suppressing the MAPK signaling pathway (p-JNK and p-p38) and regulating the Bcl-2/Bax ratio to lower ROS levels [301]. Quercitrin has been confirmed to be able to directly or indirectly weaken the differentiation of osteoclasts through its antioxidant properties [302]. Apigenin has been reported to have a certain therapeutic effect in bone diseases related to oxidative stress [154]. And the compound can induce apoptosis of rheumatoid arthritis fibroblast-like synoviocytes (RA-FLS) by mediating ROS and activating the oxidative stress factor ERK1/2 [303,304]. It further mediates apoptotic cell death through activating the apoptosis effector protein caspase-3/7, thereby exerting a pro-apoptotic effect on human RA-FLS [303,304]. Luteolin has also been proven to inhibit the production of ROS [305]. In an oxidative stress model, luteolin can protect osteoblasts from H_2_O_2_ damage by scavenging ROS and enhancing the activity of superoxide dismutase, and inhibit adipogenic differentiation by activating the AMPK signal [20]. Its protective effect against oxidative stress is further manifested in the arthritis model by inhibiting the decomposition factors of chondrocytes (MMP1 and A disintegrin and metalloproteinase with thrombospondin motifs 5) and promoting the synthesis factors (aggrecan and Col II), and maintaining cartilage homeostasis through autophagy regulation [306]. It has been reported that kaempferol can enhance the activity of antioxidant enzymes, and its oxidation metabolites (such as 2-(4-hydroxybenzoyl)-2,4,6-trihydroxy-3(2H)-benzofuranone) have significantly better antioxidant capacity than the precursors, and can achieve cell protection at extremely low concentrations [21].

#### 3.4.2. Other Active Constituents

Rensburg et al. reported that plant sterols such as β-sitosterol and its glucoside can reduce membrane lipid peroxidation [307]. β-sitosterol can treat rheumatoid arthritis by inhibiting inflammatory mediators and lipid peroxidation [308]. The use of protocatechuic acid in rat models can improve rotator cuff repair and reduce fatty degeneration [309]. Chlorogenic acid can inhibit apoptosis by reversing the generation of intracellular ROS, H_2_O_2_ concentration, and excessive mitochondrial superoxide production [229]. Chlorogenic acid can also protect osteoblasts by regulating the activation of the PI3K/AKT/Nrf2/HO-1 and p21/Nrf2/HO-1 antioxidant stress signaling pathways [175,176,310].

### 3.5. Angiogenesis Regulation

Angiogenesis constitutes an indispensable biological basis for bone defect repair, representing a highly coordinated regulatory mechanism through which living systems orchestrate tissue regeneration and functional reconstruction. This process extends beyond the provision of nutrients, serving as a central regulatory hub that maintains the dynamic equilibrium of the osteogenic microenvironment and determines the efficacy threshold of tissue engineering strategies. Within the complex cascade of bone defect healing, angiogenesis functions as a symphonic conductor, integrating multidimensional interactions among cellular behaviors, extracellular matrix deposition, and mechanical adaptation. The early establishment of vascular networks not only delivers oxygen and essential nutrients to the regenerating regions but also forms a “biological highway” for stem cell migration, with its branching architecture directly guiding the spatiotemporal distribution of osteoprogenitor cells [5,311]. Clinical evidence indicates that bone grafts lacking effective vascularization exhibit failure rates as high as 40%, whereas vascularized tissue-engineered bone constructs demonstrate more than a threefold increase in the rate of osseointegration [312]. The molecular “crosstalk” between vascular endothelial cells and osteoblasts reflects an evolutionarily conserved cooperative mechanism. Signaling molecules secreted by the neovasculature act as biological signals to activate the osteogenic differentiation potential of mesenchymal stem cells, while the three-dimensional (3D) topological structure of vascular networks provides a biomimetic scaffold for mineral deposition. This dynamic bidirectional interaction elucidates the necessity of prevascularization strategies in the repair of segmental bone defects exceeding 5 cm, as simple scaffold implantation frequently results in central necrosis due to ischemic conditions.

#### 3.5.1. Flavonoids

Bone, being a highly vascularized organ, critically depends on angiogenesis during osteogenesis, the level of which represents a potential therapeutic strategy for compromised bone repair and regeneration. Chen et al. demonstrated that systemic administration of icariin enhances angiogenesis and tendon-bone healing post-rotator cuff reconstruction, yielding superior mechanical strength [313]. Notably, while both icariin and icariside II effectively stimulate angiogenesis, icariside II exhibits stronger upregulation of VEGF [314]. NOS, produced by osteocytes, has been shown to suppress osteoclast-mediated bone resorption while stimulating osteoblast activity [315]. Icariin was found to augment nitric oxide (NO) production and endothelial NOS (eNOS) expression in human endothelial cells [316]. Icariin also produced endothelium-dependent vasorelaxation through activation of eNOS and the NO-cyclic guanosine monophosphate (cGMP) pathway, reinforcing its potential to improve local perfusion [29,30]. Mechanistically, icariin promotes osteogenic differentiation of rat bone marrow stromal cells via activation of the PI3K/AKT/eNOS/NO/cGMP/dependent protein kinase signaling cascade [317]. Concurrently, icariside II stimulates osteogenesis by enhancing inducible NOS activity and NO synthesis [318]. In vivo study using BALB/c nude mice revealed that epimedin C potently facilitates BMP-2-dependent bone formation by inducing endothelial-like differentiation of C3H/10T1/2 cells [319]. Quercetin induces bone regeneration through upregulated expression of VEGF, angiopoietin-1, basic fibroblast growth factor, and TGF-β [320]. The combination of kaempferol and metformin synergistically promotes osteogenic-angiogenic coupling via the Notch pathway, elevating expression of bone formation markers (e.g., VEGF and Runx2) while improving bone mechanical properties in OVX rats [321]. Further investigations confirmed that the coadministration of these two drugs can stimulate pro-osteogenic factors and VEGF in osteoblasts while inducing angiogenesis in human umbilical vein endothelial cells (HUVECs) through enhanced osteogenic-angiogenic coupling, with Notch pathway involvement [321].

#### 3.5.2. Other Active Constituents

β-sitosterol attenuates joint swelling and bone destruction in collagen-induced arthritis mice by suppressing VEGF-mediated synovial angiogenesis [322]. Caffeic acid phenethyl ester upregulates VEGF production in MC3T3-E1 cells and induces phosphorylation of ERK, p38, MAPK, and stress-activated protein kinase (SAPK)/JNK, all crucial for bone repair [323].

### 3.6. Antitumor Effects

Reconstruction of oncologic bone defects (e.g., post-osteosarcoma or metastatic tumor resection) requires tumor-free microenvironment verification, as residual malignant cells may disrupt regeneration through aberrant proliferation. Anti-neoplastic interventions—including surgical margin clearance, targeted therapy, or radiation—establish “biological safety zones” for bone tissue engineering. Emerging evidence indicates that certain antitumor modalities concomitantly activate stress-responsive repair mechanisms in osteoprogenitors, achieving therapeutic-regenerative synergy [324].

#### 3.6.1. Flavonoids

Icariin suppresses prostate cancer metastasis and osteolysis via blockade of the tumor-associated macrophage/chemokine (C-C motif) ligand 5/osteoclast pathway [325]. Epithelial–mesenchymal transition (EMT), a pivotal process in osteosarcoma metastasis, is potentiated by miR-194/215 cluster upregulation. Icaritin–doxorubicin combination exerts additive/synergistic antiproliferative effects on MG-63 cells, potentially through Bcl-2, caspase-3, and p21 gene regulation [326]. Icariside II diminishes osteosarcoma cell metastatic potential by targeting phospho-glycerate kinase 1 and suppressing miR-194/215-mediated EMT [327]. Icariside I and icariside II counteract cyclophosphamide-induced myelosuppression in mice by regulating hematopoietic cytokines, maintaining Bcl-2/Bax balance, and inhibiting caspase-3 expression [54]. Icariside II also suppresses HIF-1α in human osteosarcoma cells [27]. Hyperoside significantly inhibits proliferation of U2OS and MG63 osteosarcoma cells by inducing G0/G1 cell cycle arrest while impairing migration and invasion [28]. Hyperoside downregulates Wnt/β-catenin signaling and suppresses genes associated with multiple myeloma progression [328]. Apigenin inhibits osteosarcoma proliferation and invasion via Wnt/β-catenin suppression [328,329]. It attenuates EMT, stemness, and Warburg effect in SOSP-9607 cells through PI3K/AKT/mTOR modulation [328,329]. It also induces osteosarcoma apoptosis via caspase-3/8/9, Bax, and apoptosis-inducing factor pathways while suppressing xenograft growth [25].

#### 3.6.2. Other Active Constituents

Magnoflorine enhances cisplatin sensitivity and suppresses malignant phenotypes in osteosarcoma via miR-410-3p/high mobility group box 1/NF-κB axis modulation [330]. Menadione and protocatechuic acid cotreatment reduces viability, delays migration, and decreases NADPH oxidase 2 expression/ROS generation, enhancing antineoplastic efficacy against murine osteosarcoma [331].

### 3.7. Chondroprotective Effects

Cartilage plays a pivotal role in bone tissue engineering. As a biological precursor to natural bone, cartilage facilitates new bone formation through a process of staged mineralization. Its porous extracellular matrix not only supports cell migration and vascular ingrowth but also establishes a conducive microenvironment for the directed differentiation of osteoprogenitor cells. During bone regeneration, the mechanical buffering capacity of cartilage effectively distributes interfacial stress between the implant and host bone, thereby minimizing stress concentration and reducing the risk of early implant loosening. Furthermore, chondrocyte-secreted ECM components can activate osteogenic signaling pathways and promote osteoblast proliferation and ECM mineralization via intercellular crosstalk. In the development of composite scaffolds, cartilage tissue functions as a bioactive component that enhances host–tissue integration through strategic surface modifications [332]. Importantly, its degradation profile closely aligns with the rate of bone regeneration, mitigating the need for secondary surgical interventions associated with mismatched degradation kinetics in conventional biomaterials. Clinically, cartilage-based engineered constructs have demonstrated robust osteoinductive potential, offering a promising therapeutic approach for the repair of complex bone defects [333].

#### 3.7.1. Flavonoids

The osteoinductive effect of icariin in vivo can be achieved through endochondral ossification [32]. As a safe and potent anabolic agent for chondrocytes, icariin can influence chondrocyte proliferation and reduce ECM degradation [334,335]. Icariin can increase the secretion of Col I and Col II, and the abundant ECM is conducive to the integration of new cartilage and bone [336]. Additionally, Icariin has the potential to induce the proliferation and differentiation of BMSCs into chondrocytes and protect rabbit BMSCs from apoptosis induced by oxygen-glucose deprivation [337]. Interestingly, icariin can accelerate cartilage defect repair by promoting chondrogenic differentiation of BMSCs under oxygen-glucose deprivation conditions [338]. Ziadlou et al. found that epimedin C significantly promoted cartilage regeneration by increasing collagen and non-collagen matrix proteins, demonstrating a clear anabolic effect [339]. Kaempferol has been reported to induce chondrogenesis in ATDC5 cells through activating the ERK/BMP-2 signaling pathway [340].

#### 3.7.2. Other Active Constituents

Chlorogenic acid can prevent chondrocyte apoptosis caused by thiabendazole by down-regulating the mRNA and protein expression of Bax/caspase-3 and up-regulating the expression of Bcl-2 (under the action of the CD147 signaling axis), thereby treating tibial cartilage dysplasia [341]. And it can positively treat tibial dyschondroplasia caused by mancozeb by regulating the expression of caspase and beclin 1, as well as controlling the degradation of ECM [342]. Caffeic acid has a positive effect on alleviating adjuvant-induced arthritis, which can effectively down-regulate NF-κB, possibly explaining the observed inhibition of downstream effectors chitinase-3-like protein 1, IL-1β, and MMP-9, thereby reducing cartilage degeneration and joint damage [74,343,344].

### 3.8. Antibacterial Effects

Pathogen infection, such as that caused by *Staphylococcus aureus*, remains a critical challenge in the clinical management of nonunion and bone defects, necessitating effective eradication. These pathogens not only directly impair osteoblast function but also trigger inflammatory responses—such as the release of TNF-α—that disrupt bone matrix deposition and vascularization [345]. Infection-induced immune evasion mechanisms, including the formation of polysaccharide-protein complex barriers, along with the growing prevalence of antimicrobial resistance, demand a multifaceted therapeutic approach [346]. This includes surgical debridement, localized sustained-release antibiotic systems, and surface modification of biomaterials (e.g., silver ion coatings) to reestablish a conducive microenvironment for bone regeneration [347,348]. *Epimedium*, a classical traditional Chinese medicinal herb renowned for its kidney-tonifying and bone-strengthening properties, is increasingly being explored in bone tissue engineering beyond its traditional role in osteogenesis promotion, now extending to anti-infective functionalities [167]. Current research focuses on harnessing the multi-modal pharmacological activities of its bioactive constituents—particularly flavonoids such as icaritin—to engineer an integrated “anti-inflammatory, antibacterial, and pro-osteogenic” microenvironment for enhanced bone repair.

Under conditions of *Porphyromonas gingivalis* infection, icariin can downregulate the EphA2-RhoA signaling pathway, thereby enhancing osteogenic differentiation and bone regeneration, with 10 μM icariin showing the most pronounced effect [167]. However, Liu et al. confirmed icariin exhibits limited antibacterial activity against bacteria associated with peri-implantitis [349]. This research finding was partially corroborated by Peris et al. [350]. They demonstrated that icariin alone exhibited no antimicrobial activity against clinical isolates of *Staphylococcus aureus* or methicillin-resistant *Staphylococcus aureus* [350]. Nevertheless, it is noteworthy that icariin enhanced the antibacterial efficacy of amoxicillin-clavulanate and ampicillin, while no synergistic effect was observed when combined with vancomycin [350]. Moreover, oral administration of luteolin alleviated maxillary alveolar bone resorption in murine periodontitis induced by *Porphyromonas gingivalis* infection [351]. Furthermore, a study of Gao et al. demonstrated that the combination therapy of azithromycin and kaempferol was more effective in countering azithromycin-resistant *Staphylococcus aureus* bacterial growth by suppressing the phosphorylation of ERK 1/2 and SAPK [352]. In fact, the antibacterial activity of flavonoids primarily arises from the multi-target synergistic effects of their C6-C3-C6 skeleton and associated substituents. The hydrophobic core structure can insert into the lipid bilayer of bacterial cell membranes, while the phenolic hydroxyl groups on the B ring—particularly the catechol moiety—can disrupt membrane integrity through hydrogen bonding, resulting in leakage of intracellular contents [353]. Furthermore, the ortho-dihydroxyl groups and the carbonyl group at the 4-position form a potent metal-chelating system that deprives bacteria of essential metal ions and catalyzes the generation of reactive oxygen species, thereby inducing oxidative stress [353]. Additionally, the conjugated planar structure of the molecule—such as the presence of a C2=C3 double bond—enables effective intercalation into and inhibition of the active sites of key bacterial enzymes (e.g., DNA gyrase and fatty acid synthase), interfering with nucleic acid replication and metabolic pathways [353]. Therefore, the antibacterial mechanism of flavonoids is attributed to the combined effects of hydrophobic interactions, membrane disruption, metal ion chelation, and enzymatic inhibition. Figure 3 illustrates the key biological mechanisms by which phytochemicals isolated from *Epimedium* may promote bone regeneration at defect sites.

### 3.9. Comparative Analysis of Active Constituents’ Bone Functions

Icariside is one of the bioactive constituents of *Epimedium*. It is demonstrated that following oral administration of icariin, various metabolites such as icariside I, icariside II, and icaritin are generated through degradation by intestinal flora [54]. These metabolites tend to accumulate at higher concentrations in tissues such as the kidneys, liver, and spleen [54]. Pharmacokinetic studies reveal icariside II’s superior bioavailability and absorption kinetics compared to icariin [354,355], with icariside I exhibiting enhanced absorption [54]. Data from rats treated with 30 mg/kg of oral icariside II showed a terminal elimination half-life of 135 min and an absolute bioavailability of 4.1%. Tissue distribution studies in rats show rapid icariside II dispersion to skeletal and muscular systems following intravenous administration, with minimal testicular accumulation and undetectable cerebral penetration [356,357]. Notably, icariside II exhibits both greater biological activity and a longer half-life compared to icariin, and it is also characterized by rapid absorption [354,355]. Furthermore, experimental findings indicate that the absorption efficiency of icariside I surpasses that of icariin. Although the natural abundance of icariside II in *Epimedium* (<0.15%) is lower than icariin, it demonstrates a broader spectrum of pharmacological activities [358,359].

There is still controversy over the strength of the in vivo and in vitro biological activities of the active constituents of *Epimedium*. In the regulation of bone regeneration, Wu et al. reported that although the osteogenic activity of icaritin in vitro is lower than that of recombinant human BMP-2, icaritin at a concentration of 1 × 10^−6^ M exhibits a stronger effect than icariin [133]. Notably, icariin can achieve a bone-enhancing effect comparable to that of BMP-2 (50 μM) at a concentration of 30 μM [360].

Some researchers have pointed out that the effects of various chemical components in *Epimedium* on the treatment of osteoporosis vary in strength, with the following rank order: icariin > icariside II > epimedin A > epimedin B > epimedin C [361]. Huang et al. demonstrated through experimental studies that icaritin and its glycosides can promote the proliferation of osteoblasts in vitro in a dose-dependent manner, with maximal effects observed at a concentration of 1 × 10^−6^ mol/L [14]. Furthermore, the stimulatory effect of icaritin exceeds that of icariin and icariside II, although it remains lower than that of genistein [14]. Overall, icaritin has the best promoting effect. Research has demonstrated that icaritin and its glycosides can inhibit osteoclast formation. Moreover, at a concentration of 1 × 10^−5^ mol/L, icaritin exhibits efficacy comparable to that of genistein (46.1 ± 7.1% vs. 46.8 ± 10.3%) [14]. Treatment of bone marrow macrophages with icariin and icaritin can inhibit the differentiation of these cells into osteoclasts induced by M-CSF/RANKL. Researchers suggest that osteoclast precursors may represent another cellular target for icariin and icaritin, whereas icariside II does not exhibit this activity [14]. Moreover, icaritin and its glycosides can reduce the absorption of bone by osteoclasts. In this regard, the three flavonoid glycosides (i.e., icariin, icaritin, and icariside II) are comparable to genistein [14]. In addition, it was observed that icaritin and its glycosides can reduce the number of actin rings in osteoclasts, thereby reducing their size, but have no effect on the number of osteoclasts [14]. The order of inhibitory effect is icaritin > icariside > icariin, and the inhibitory effect of icaritin is stronger than that of genistein, with values representing 35.2 ± 21.1% and 57.5 ± 14.3% of the control, respectively [14]. Also, icaritin and its glycosides can reduce the generation of superoxide anions in osteoclasts, with icaritin exhibiting the most significant effect, thereby contributing to the elimination of ROS [23]. This mechanism may attenuate osteoclast formation and reduce bone resorption [14,362,363,364].

Estrogen plays a pivotal role in bone tissue engineering by regulating bone homeostasis through multiple mechanisms and serves as a critical effector molecule in promoting bone regeneration [365]. The core mechanism of its action can be summarized as follows: (1) Estrogen directly activates osteoblasts by binding to ESRα, upregulating the expression of osteogenic transcription factors, promoting collagen synthesis and ECM mineralization, and simultaneously inhibiting osteoblast apoptosis to extend their functional lifespan [366,367]. (2) Estrogen can modulate the RANKL/OPG signaling axis to block osteoclast activation and significantly suppress bone resorption [368]. (3) Estrogen interacts synergistically with the growth factor network, stimulating insulin-like growth factor-1 secretion and activating the Wnt/β-catenin pathway, which enhances the proliferative and differentiative capacity of osteoblasts and establishes a positive feedback loop for bone formation [369]. (4) Estrogen contributes to the maintenance of bone microstructural integrity through the regulation of calcium metabolism—including enhancing vitamin D activity—thus preventing trabecular bone deterioration associated with postmenopausal osteoporosis [370]. In the context of bone tissue engineering, estrogen-mediated mechanisms offer precise targets for scaffold design and optimization of cell culture protocols, thereby significantly improving bone regeneration efficiency by balancing bone formation and resorption. The estrogenic effects of various flavonoids from *Epimedium* exhibit considerable variability. Studies have demonstrated that the estrogenic activity of *Epimedium* may be mediated by icaritin and demethyl icaritin, with the mechanism of action potentially involving ESRs or other key regulatory proteins [371]. Icariin can be metabolized into icaritin and demethyl icaritin, a metabolic transformation that may account for its selective estrogenic activity via ERα [193,372,373]. Wong et al. evaluated the estrogenic effects in rat serum using ESRα- and ESRβ-based bioassays and reported a potency order of demethyl icaritin > icaritin > icariside I > icariside II [374]. Additionally, the results indicated that icariin may lack intrinsic estrogenic activity, despite being the primary flavonoid glycoside in *Epimedium* extracts [374]. Liu et al. also reported that icariin exhibited little to no estrogenic activity, as determined by in vitro estrogenic activity assessment using human MCF-7 breast cancer cells in the E-screen assay [375]. Nevertheless, He et al. discovered that when administered peripherally, the central pharmacological effects of icariside II were significantly stronger than those of icaritin [376]. It is evident that following oral administration, icariin may exert its biological effects primarily through its metabolic derivatives. This hypothesis has been further supported by findings from multiple research studies [81,371,374,377,378]. Moreover, icaritin and icariside II display higher musculoskeletal penetration efficiency compared with icariin, with icariside II demonstrating a distinct advantage in tissue permeability [379]. However, with regard to reversing glucocorticoid-induced inhibition of bone formation, icaritin shows superior efficacy compared with icariin and icariside II [380].

### 3.10. Network Pharmacology

#### 3.10.1. Network Pharmacology Analysis

The structures and identifiers of icariin, icariside II, and icaritin were obtained from PubChem. We entered the compound names, including icariin, icaritin, and icariside II into the search bar, then we selected the best compound match for each, and obtained their SMILES codes. Drug-likeness and physicochemical properties were evaluated using SwissADME (accessed on 2 September 2025) [381]. Putative human protein targets were retrieved from SwissTargetPrediction (accessed on 4 September 2025) [382] with the organism restricted to Homo sapiens. Targets associated with the 53 bone-related conditions were retrieved from GeneCards (accessed on 4 September 2025) [383], OMIM (accessed on 4 September 2025) [384], and TTD (accessed on 4 September 2025) [385] using the exact disease terms listed in Supplementary Table S1. Genes retrieved from GeneCards with a query-specific Relevance Score ≥ 1.0 were retained. For OMIM and TTD, all gene entries linked to the prespecified disease terms in OMIM and all disease-associated therapeutic targets retrieved from TTD were retained without applying database-specific score or evidence-level cutoffs. The retrieved identifiers were standardized to the corresponding human gene symbols and UniProtKB accession numbers using the UniProt ID Mapping service (accessed on 5 September 2025) [386]. Records that could not be mapped to Homo sapiens entries were excluded, and duplicate gene entries were removed before dataset integration. Compound-associated and disease-associated target sets were intersected in Venny 2.1.0 (BioinfoGP, CNB-CSIC; accessed on 7 September 2025) using exact approved gene symbols (Supplementary Table S2). Protein–protein association analysis was performed using STRING v12.0, with the organism restricted to Homo sapiens (NCBI taxon ID: 9606). The default full STRING network was used, and associations with a combined confidence score ≥ 0.400 (medium confidence) were retained. No additional interaction partners were added. Disconnected nodes were hidden from the network visualization. The network was visualized using Cytoscape v3.10.3. Betweenness centrality (BC), closeness centrality (CC), and degree centrality (DC) were calculated using the CytoNCA v2.1.6 plugin. Hub targets were defined as nodes whose BC, CC, and DC values were each greater than the corresponding median calculated across all nodes in the original PPI network (BC > (actual median), CC > (actual median), and DC > (actual median)) and at the same time all three criteria had to be satisfied simultaneously (Supplementary Table S2). We performed functional enrichment analysis with the DAVID Functional Annotation Tool (DAVID Knowledgebase v2025q1, accessed on 7 September 2025), using the default genome-wide Homo sapiens gene set as the background. KEGG pathway annotations were drawn from release 112.0, which was incorporated into this version of the DAVID Knowledgebase. We did not modify the analysis parameters. The Functional Annotation Chart therefore used DAVID’s default criteria of at least two genes per term and an EASE score ≤ 0.1. We applied no additional cutoff based on the Benjamini–Hochberg-adjusted *p* value or FDR. From the returned KEGG terms, we manually selected those related to bone formation and resorption, skeletal development and remodeling, mineralization, and osteoblast or osteoclast differentiation and function for focused presentation and interpretation (Supplementary Table S3). The heatmap was plotted using https://www.bioinformatics.com.cn (accessed on 10 September 2025), an online platform for data analysis and visualization. A dot plot shows the results of the KEGG pathway enrichment analysis. The horizontal axis represents the gene ratio, while the vertical axis represents the enriched pathway name. The color scale indicates different thresholds of the *p*-value, and the size of the dot indicates the number of genes corresponding to each pathway [387]. We treated these associations as hypothesis-generating rather than as evidence of causal mechanisms.

#### 3.10.2. Molecular Docking

To assess the key interactions predicted by network pharmacology, we performed molecular docking. Hub targets were screened from the PPI network using Cytoscape, retaining only nodes whose NAC, BC, CC, and DC values exceeded the respective median values (Supplementary Table S2). Then, the selected targets were docked with three bioactive compounds: icariin, icaritin, and icariside II. For the “Preparation of ligands and proteins” procedure: the three-dimensional structures of the selected bioactive compounds were downloaded from PubChem in SDF format (Supplementary Table S3). Crystal structures of the hub target proteins were retrieved from the Protein Data Bank (PDB). Protein structures were processed in PyMOL (v2.1.0) by removing water molecules and co-crystallized small-molecule ligands. Hydrogen atoms and charges were then added using AutoDockTools (v1.5.6), and the prepared proteins were saved in PDBQT format. For the “Docking procedure and parameters” procedure: using each processed protein as a receptor and its corresponding active compound as a ligand, molecular docking was performed with the built-in Vina 2.0 engine in PyRx. For each receptor, we defined the docking search space using a grid box centered on the selected binding site. The grid-center coordinates and box dimensions are listed in Supplementary Table S3. Vina generated predicted binding affinities and docking poses for each ligand-receptor pair. We visualized the docking poses in PyMOL (v2.1.0; accessed on 18 September, 2025). We interpreted more negative affinity values (kcal/mol) as indicating stronger predicted ligand-receptor binding.

#### 3.10.3. Network Pharmacology-Based Analysis of Key Flavonoid

Compared with other constituents, flavonoids derived from *Epimedium* have been more extensively studied and applied. Multiple studies have demonstrated a well-established metabolic relationship among icariin, icaritin, and icariside II [48,49,50,51,380]. Consequently, we conducted network pharmacology-based predictions for these three flavonoids and analyzed the current research landscape. As shown in Figure 4A, the physicochemical properties of icariin, icaritin, and icariside II were retrieved and evaluated using the SwissADME database [381]. Potential target proteins of these compounds were identified using SwissTargetPrediction database. These targets were subsequently cross-referenced with 53 disease-associated targets related to bone disorders, as predicted by the GeneCards, Online Mendelian Inheritance in Man, and Therapeutic Target Database [383,385,388]. The intersecting targets were imported into the Search Tool for the Retrieval of Interacting Genes/Proteins database to obtain a protein–protein interaction network diagram and visualized using Cytoscape software to further clarify the core targets. Core targets were subsequently subjected to Kyoto Encyclopedia of Genes and Genomes (KEGG) pathway enrichment analysis using the Database for Annotation, Visualization, and Integrated Discovery [389,390,391]. Finally, molecular docking studies were performed with AutoDock Vina to evaluate the binding affinities between these flavonoids and their corresponding core targets [392,393].

The relative molecular weights follow the order icariin > icariside II > icaritin (Figure 4E), consistent with gut microbiota-mediated deglycosylation: hydrolysis of the glucose moiety of icariin generates icariside II, which can be further converted to icaritin and desmethylicaritin [79,378]. The partition coefficient reflects lipophilicity, with higher values indicating greater lipophilic character and reduced hydrophilic properties. Optimal gastrointestinal absorption requires a balance between lipophilicity and hydrophilicity, as excessive values in either property are suboptimal. Consequently, icaritin demonstrates the highest gastrointestinal absorption efficiency among the three compounds. Bioavailability predictions further reveal that icaritin and icariside II exhibit substantially higher bioavailability compared with icariin.

The results of the network pharmacology analysis are shown in Figure 4. Given the recognized metabolic interplay among icariin, icaritin, and icariside II, extensive research has explored their distinct effects on bone metabolism, yet their exact molecular mechanisms require further clarification. In vitro evidence demonstrated that icaritin most effectively promoted osteoblast proliferation and differentiation compared to icariin and icariside II [74]. Additionally, the inhibitory effect on osteoclastogenesis ranked in the order of icaritin > icariside II > icariin. Regarding estrogen-like activity, icaritin again surpassed icariside II, while icariin lacked measurable intrinsic estrogenic function [14,133,374,375]. This pharmacological profile aligned perfectly with our network analysis, which pinpointed ESR1 as a core target for icariside II and both ESR1 and ESR2 for icaritin, while excluding these receptors for icariin. These functional discrepancies in estrogen-like activity primarily originate from structural differences (Figure 2), specifically the extent of deglycosylation and spatial steric hindrance, which fundamentally alter receptor binding affinity.

Icariin yielded 25 core targets, and KEGG enrichment analysis revealed 48 bone pathways related to bone metabolism (Figure 4C), among which one core target-F2 and 29 pathways were not identified within the databases or search framework used in this study. Molecular docking demonstrated stable predicted binding (binding energy < −5.0 kcal/mol) between icariin and 17 core targets—AKT1, Bcl-2, HER1, coagulation factor II (F2), heat shock protein 90 kDa alpha A1 (HSP90AA1), integrin beta 3, nuclear factor of kappa light polypeptide gene enhancer in B-cells 1, peroxisome proliferator-activated receptor-γ, protein kinase cAMP-dependent catalytic alpha (PRKACA), protein kinase C alpha (PRKCA), protein kinase C beta, prostaglandin-endoperoxide synthase 2 (PTGS2), protein tyrosine phosphatase non-receptor type 1 (PTPN1), v-rel avian reticuloendotheliosis viral oncogene homolog A, serpin peptidase inhibitor clade E member 1 (SERPINE1), TNF-α, and tumor protein 53 (TP53)—that are enriched in these pathways, suggesting their potential significance in the regulation of icariin-mediated bone metabolism.

Icariside II analysis identified 21 core targets and 41 bone-related pathways (Figure 4B), among which six core target-PTPN1, ABL1, CTNNB1, CHEK1, SIRT1, FASN and 35 pathways were not identified within the databases or search framework used in this study. These core targets exhibited strong docking affinities (binding energy < −5.0 kcal/mol) with icariside II, suggesting their potential involvement in the osteomodulatory effects of this compound. For instance, SIRT1 is a key regulator of cellular-senescence-associated pathways [394]. In osteoblast progenitors, SIRT1 deficiency impairs cortical bone formation, and the age-associated decline in SIRT1 activity may contribute to progressive bone-mass loss [395]. The above analysis suggests that icariside II may be a promising candidate molecule for intervening in age-related osteoporosis compared to icariin, and its specific mechanisms against skeletal aging require further confirmation through future in vitro and in vivo experiments.

Analysis of icaritin identified 17 core targets and 16 bone-related pathways, among which one core target-CTNNB1 and 12 were not identified within the databases or search framework used in this study. Eleven core targets (AKT1, BCL2, CTNNB1, HER1, HER2, ESR1, ESR2, glycogen synthase kinase-3 beta, HSP90AA1, HSP90AB1, and microtubule-associated protein tau) demonstrated high-affinity binding (binding energy < −5.0 kcal/mol) with icaritin, indicating their potential critical role in the regulation of bone metabolism by icaritin. Notably, CTNNB1 was identified as a core target of icariside II and icaritin, but not of icariin. CTNNB1 encodes β-catenin, a key effector molecule in the canonical Wnt signaling pathway involved in regulating osteoblast differentiation and bone formation [395,396]. Therefore, it can be inferred that the regulatory involvement of the Wnt/β-catenin signaling pathway may provide potential mechanistic insights into why icaritin exhibits stronger osteogenic differentiation activity compared to icariin. Further literature review revealed that icaritin demonstrates the strongest effects among the three compounds in promoting osteogenesis and inhibiting osteoclastogenesis [14]. This is closely associated with its metabolic pathway, specifically the conversion of icariin to icariside II and subsequently to icaritin, which constitutes a progressive deglycosylation process. This process involves the sequential removal of hydrophilic glycosyl groups, a gradual reduction in molecular weight, and the exposure of more active hydroxyl groups. Consequently, this structural modification increases the lipophilicity of icaritin and significantly enhances its cell membrane permeability, thereby enabling it to penetrate cells more easily to exert its biological activities.

The above content systematically reveals the evolutionary process of icariin in terms of “structure-metabolism-target-efficacy.” By identifying novel targets such as SIRT1 and CTNNB1, it provides a microscopic mechanistic hypothesis for how deglycosylation enhances bioavailability and promotes osteogenic activity, overcoming previous limitations that were confined to macro-level efficacy comparisons. This advancement significantly contributes to a comprehensive understanding of the pharmacological basis of icariin. However, these mechanistic hypotheses still require further validation through pharmacokinetic tracking, interventions targeting specific pathways, and functional experiments both in vitro and in vivo.

## 4. Applications in Bone Tissue Engineering

*Epimedium*, a traditional Chinese medicinal herb, promotes bone regeneration and plays multifaceted roles in bone tissue engineering. Its core bioactive components facilitate bone metabolic homeostasis through multi-target modulation, enhancing its therapeutic potential. Current applications predominantly rely on biomaterial-based localized delivery systems, with emerging studies reporting synergistic therapeutic effects when its extracts are combined with stem cell therapy, resulting in bone healing rates over 70% in animal models of critical-sized bone defects [381]. Through the in-depth integration of materials science, engineering, and traditional Chinese medicine, *Epimedium* holds promise as a crucial component in innovative “bioactive scaffold + endogenous repair” systems for bone regeneration, potentially advancing the transition of bone tissue engineering from replacement therapy to functional reconstruction (Figure 5 and Table 2).

### 4.1. Hydrogels

Hydrogels are three-dimensional, crosslinked polymeric networks capable of absorbing and retaining large quantities of water or biological fluids, thereby enabling controlled release of encapsulated therapeutic agents [397,398,399]. Due to their tunable properties and diverse fabrication methodologies, hydrogels have been widely utilized in the field of biomedical engineering [400]. Among various research efforts aimed at enhancing drug delivery efficacy while minimizing adverse effects, hydrogel-mediated sustained drug release has emerged as a highly promising strategy [401].

Chen et al. encapsulated icariin-loaded zinc–aluminum layered double hydroxide nanosheets into a gallic acid-modified hydroxy butyl chitosan (CTS) hydrogel to develop a tailored hydrogel system [401]. This system achieves sequential antibacterial, anti-inflammatory, and remineralization functions through programmed spatiotemporal dynamic sequences [401]. It synergistically enhances the therapeutic effects of icariin, thereby effectively promoting the regeneration and repair of chronic periodontitis accompanied by bone defects [401]. A novel clinical strategy for the treatment of severe bone and cartilage defects has been developed through the introduction of hydroxyethyl methacrylate (HEMA) groups and the functionalization of thermosensitive hydrogels with HEMA moieties [402]. This system employs supercritical carbon dioxide to achieve controlled release of poly(N-vinyl caprolactam) (PNVCL)@icariin nanoparticles for icariin delivery, while maintaining the optimal compositional characteristics of the ECM [402]. Zhu et al. developed an integrated system that combines a 3D-printed titanium alloy scaffold with a thermosensitive chitosan hydrogel, thereby facilitating localized bone regeneration and enhanced osseointegration through the delivery of icariin [403]. The scaffold exhibits controlled icariin release kinetics, demonstrates exceptional biocompatibility, and effectively promotes the proliferation and osteogenic differentiation of BMSCs [403]. Moreover, Song et al. coated titanium surfaces, which were pretreated with phase transited lysozyme, with icariin-loaded hyaluronic acid (HA)/CTS multilayer films [404]. This configuration exhibits sustained icariin release characteristics, effectively promoting osteogenic differentiation of osteoblasts in vitro and enhancing early osseointegration in vivo [404]. Fan et al. developed an icariin-loaded CTS/hydroxyapatite composite hydrogel where the release kinetics of icariin were governed by the simultaneous degradation of both CTS and hydroxyapatite matrices [405]. The design enabled the sustained release for over 90 days while effectively stimulating ALP activity in bone marrow stromal cells and promoting mineralized nodule formation [405]. In the study of Shao et al., a thermosensitive icariin/CTS/β-glycerophosphate hydrogel was engineered to modulate inflammatory responses, angiogenesis, and osteogenic factor expression, demonstrating potent infection control efficacy in a canine model of infectious bone defect [406]. Researchers proposed that the combined application of icariin-conditioned serum with thiolized CTS could mitigate cartilage degeneration and subchondral bone remodeling abnormalities in rabbit models of knee osteoarthritis [407]. Fu et al. constructed an injectable sodium alginate (SA) hydrogel by encapsulating icariin, titanium carbide powder (Ti_3_C_2_T_x_), and doxorubicin (DOX) [408]. The incorporation of icariin attenuated DOX-induced inhibition of BMSCs proliferation and differentiation, thereby conferring the hydrogel with robust antitumor efficacy while significantly reducing osteogenic toxicity [408]. Similarly, a composite hydrogel was developed based on a hydrogel matrix composed of Pluronic F127 (PF127) and Pluronic F68 (PF68), co-loaded with icaritin and DOX [409]. The incorporation of icaritin not only decreased the required dosage of DOX and its associated toxic side effects, but also promoted osteogenic differentiation, thereby facilitating osteosarcoma treatment and supporting bone regeneration [409]. Further, Qian et al. developed a unique cell-infiltratable and injectable gelatin hydrogel utilizing a high degree of acryloyl-modified β-cyclodextrin (β-CD) [13]. The hydrophobic cavities within β-CD in the hydrogel were employed for the delivery of icaritin [13]. The hydrogel demonstrated sustained release of icaritin over a two-week period, promoting stem cell differentiation while reducing the adverse effects associated with high-dose drug administration [13].

Ginkgetin has been shown to enhance autophagy and suppress inflammatory responses [410,411]. Capitalizing on this advantage, Chen et al. developed a novel MMP13-responsive hydrogel nanoplatform capable of orchestrating the controlled release of ginkgetin and rapamycin to target distinct stages in the progression of intervertebral disc degeneration [412]. This strategy effectively modulates inflammation and autophagy in degenerative disc pathology [412]. A magnoflorine-loaded HA hydrogel significantly enhance subchondral bone regeneration by increasing bone volume fraction, trabecular number, and connectivity density while reducing the degree of anisotropy [413]. Furthermore, local administration of magnoflorine-loaded HA hydrogel enables effectively prevented osteoarthritis progression following anterior cruciate ligament rupture [414]. Mohammadi et al. employed a freeze-drying technique to fabricate a composite scaffold through crosslinking of gelatin, CTS, and HA, with the incorporation of diatoms and β-sitosterol [415]. This scaffold enhances cellular adhesion, promotes ECM mineralization, and stimulates osteoblast proliferation, while simultaneously facilitating angiogenesis via β-sitosterol and exerting anti-inflammatory properties [415]. Lee et al. utilized nanoclays as nanoscale crosslinkers to develop a nanoclay-reinforced caffeic acid-modified CTS hydrogel bone sealant, demonstrating an enhanced bone regeneration [416]. Just recently, Gao and colleagues functionalized poly (xylitol succinate) with caffeic acid and citric acid, followed by integration with magnesium oxide to fabricate a multifunctional injectable hydrogel possessing intrinsic antagonistic properties against the TLR4 and myeloid differentiation factor 2 complex [417]. This innovative hydrogel exhibits potent anti-inflammatory and antibacterial properties. And in murine models of periodontitis, the hydrogel not only demonstrated regenerative efficacy but also displayed therapeutic potential in reducing inflammatory cytokine levels, suppressing key periodontopathogenic bacterial species, and maintaining subgingival microbial homeostasis [417]. Palaniraj et al. fabricated a chlorogenic acid-loaded antibacterial porous nanogel based on calcium phosphate-CTS nanocomposites [418]. This nanostructured matrix, formed through ionic gelation of calcium phosphate nanoparticles acting as crosslinking agents, exhibits controlled drug release properties and pronounced antibacterial effects, showing potential in promoting the differentiation of hDPSCs [418]. Furthermore, Fang et al. developed a poly-methylacrylamide hydrogel encapsulating neochlorogenic acid and BMSCs [419]. The hydrogel effectively mitigates oxidative stress-induced damage to stem cells, reduces apoptosis rates, and enhances the survival of transplanted stem cells, thereby offering a promising therapeutic strategy for intervertebral disc degeneration [419].

Although hydrogels offer numerous advantages as sustained-release carriers for the active constituents of *Epimedium*, their limitations warrant careful consideration. Firstly, some of *Epimedium*-derived bioactive compounds exhibit poor solubility and low permeability, leading to non-uniform drug loading and compromising the synergistic effects of multi-component formulations due to disruption of the sustained-release mechanism. Secondly, conventional hydrogels are highly sensitive to environmental conditions; swelling of the hydrophilic polymer network in vivo causes drug release kinetics to deviate from zero-order behavior, resulting in an initial burst release or delayed release in later stages, thereby undermining therapeutic efficacy stability. Thirdly, synthetic materials (e.g., polyacrylic acid) may provoke inflammatory responses or immune rejection, whereas natural polymers (e.g., gelatin) often lack sufficient mechanical strength and are susceptible to microbial degradation. Additionally, implantable hydrogel systems typically require surgical intervention, and the long-term toxicity of degradation byproducts (such as lactic acid) remains unclear. Fourthly, fabrication processes are often complex—requiring techniques such as high-pressure homogenization or graft copolymerization—entailing high production costs and inconsistent batch-to-batch reproducibility. Furthermore, optimizing process parameters for multi-component self-assembly technologies remains challenging, hindering compliance with clinical standardization requirements.

### 4.2. Organic Scaffolds

In bone tissue engineering, organic scaffold materials demonstrate notable advantages and have consistently garnered significant attention. Their exceptional biocompatibility—exemplified by collagen and CTS, which mimic the structure of the natural ECM—facilitates cell adhesion, proliferation, and differentiation. Additionally, their tunable degradation rates (e.g., those of polylactic acid (PLA) and polycaprolactone (PCL)) can be aligned with the pace of bone regeneration, thereby eliminating the need for secondary surgical interventions. Furthermore, their highly customizable architectures enable precise control over porosity (>70%) through 3D printing, enhancing vascularization and nutrient transport. Mechanical properties can be further improved through crosslinking or the incorporation of inorganic phases such as hydroxyapatite. Finally, their functional versatility supports the incorporation of bioactive agents, including growth factors (e.g., BMP-2) and therapeutic drugs, to achieve synergistic osteogenic and antibacterial effects. Moreover, certain materials, such as HA, possess inherent angiogenic properties, thereby offering multidimensional support for effective bone regeneration. For instance, Xia et al. developed a novel icariin-delivering porous poly (3-hydroxybutyric acid) scaffold using solvent casting and salt leaching methods [420]. The scaffold exhibited a high porosity of 88.80% and possessed appropriate mechanical properties for three-dimensional cell culture [420]. It significantly promoted the proliferation of human osteoblast-like MG-63 cells and preosteoblastic MC3T3-E1 cells, with the formulation containing 0.1% (*w*/*w*) icariin showing the most pronounced osteogenic effect [420]. Park et al. investigated the osteogenic potential of icariin-loaded 3D-printed PCL scaffolds and demonstrated their efficacy in accelerating alveolar cleft reconstruction [421]. And Gao et al. developed a stem cell-targeted DNA tetrahedron platform (PolyApt) by conjugating Apt19s aptamer to DNA tetrahedrons [422]. Subsequently, icariin and epigallocatechin gallate (EGCG) were intercalated into the double-stranded regions of PolyApt to form PolyApt@icariin and PolyApt@EGCG complexes [422]. A degradable gradient scaffold was then engineered by encapsulating PolyApt@icariin and PolyApt@EGCG into the upper and lower layers of a gelatin methacryloyl and polyethylene glycol diacrylate matrix, respectively [422]. This design synergistically enhances the recruitment, protection, and directed differentiation of endogenous stem cells while promoting accelerated regeneration of osteochondral defects [422]. What is particularly noteworthy is that Li et al. delivered icariin in the form of ECM on a small intestinal submucosa (SIS) scaffold following icariin pre-treatment of osteoblasts [423]. Compared with direct delivery of icariin on SIS scaffold, the SIS scaffold functionalized with icariin-derived ECM exhibited superior osteoconductivity, enhanced cell adhesion, spreading, survival, and proliferation, while not compromising osteoinductivity [423].

### 4.3. Inorganic Scaffolds

Calcium phosphate cement (CPC) is an outstanding bone substitute material due to its porous structure, favorable mechanical strength, and excellent biocompatibility [424]. They are particularly suitable for clinical applications requiring rapid mechanical stability, such as vertebroplasty. Importantly, by mimicking the inorganic composition of natural bone, CPC can promote mineralization and integration with surrounding bone tissue, serving as a scaffold to support cell adhesion, proliferation, and osteogenic differentiation [425,426]. Through hydration reactions, they can form a bone-like apatite structure and undergoes gradual resorption and replacement by new bone tissue, thereby avoiding long-term foreign body retention [427]. Moreover, CPC can be combined with biomaterials such as alginate to serve as a stem cell carrier, enhancing cellular protection and collagen synthesis, or functionalized with drugs and growth factors to enable synergistic therapeutic effects [428]. Furthermore, their injectable nature and radiopacity significantly broaden their applicability in minimally invasive surgical procedures [429,430]. These characteristics render CPC a highly effective carrier for the active ingredients derived from Chinese herbal medicines. CPC scaffolds loaded with icariin can accelerate the repair and regeneration of damaged bone tissue in OVX rats [431]. Luo et al. successfully incorporated icariside II into CPC scaffolds, which was demonstrated to promote bone regeneration by enhancing the proliferation and survival of BMSCs in vivo [432]. It has also been reported that CPC scaffolds loaded with luteolin and incorporating tantalum nanoparticles significantly enhance osteogenic differentiation in vitro and bone formation in vivo through the synergistic effects of Ta^2+^ and luteolin [433]. More interestingly, Song et al. fabricated a strontium (Sr)-doped biphasic calcium phosphate (BCP) scaffold incorporating a self-crosslinked icariin network [434]. Through the synergistic action of released icariin and Sr^2+^, the scaffold enhanced osteogenesis and attenuated osteoclast activity [434].

In bone tissue engineering, although β-tricalcium phosphate (β-TCP) and CPC are both classified as calcium phosphate-based biomaterials, they differ significantly in terms of material morphology and the underlying mechanisms of osteogenesis [435]. CPC is an injectable paste that sets in situ via a hydration reaction under physiological conditions upon mixing a powdered component with a liquid phase [435]. Its key advantages include excellent adaptability to irregularly shaped bone defects, a relatively slow initial degradation rate, and its capability to serve as a carrier for therapeutic agents such as drugs or growth factors, thereby enabling precise defect filling and controlled release [435]. In contrast, β-TCP is a pre-sintered porous ceramic scaffold characterized by a stable three-dimensional architecture, which primarily provides mechanical support and facilitates cell migration and infiltration through osteoconductive guidance [435]. It exhibits a faster degradation profile compared with CPC and is primarily designed to promote bone conduction [435]. Naturally, efforts to utilize β-TCP as a carrier for bioactive components derived from Chinese herbal medicines have persisted continuously. Lin et al. innovatively fabricated an icaritin-functionalized β-TCP composite scaffold using cryogenic rapid prototyping technology, which effectively promoted neo-osteogenesis and angiogenesis while enhancing implant-bone integration [436].

Except for the CPC and β-TCP, bioactive glass is another key material in the field of bone tissue engineering, characterized by a well-established and multifaceted mechanism of action. As an excellent osteoconductive scaffold, its three-dimensional interconnected porous structure provides physical pathways for cell migration, vascular ingrowth, and nutrient transport [437]. More importantly, upon implantation in vivo, a layer of bone-like apatite rapidly forms on its surface through reactions with physiological fluids [437]. This enables the material to establish a strong biological bond with host bone, ensuring long-term implant stability, while simultaneously releasing bioactive ions such as silicon (Si^4+^) and calcium (Ca^2+^) during gradual degradation [437]. These ions have been shown to upregulate the expression of osteogenesis-related genes, directly stimulate osteoblast proliferation and differentiation, and enhance collagen synthesis and mineralization in the extracellular matrix, thereby actively promoting and accelerating de novo bone formation [437]. Furthermore, bioactive glass can serve as an ideal carrier for stem cells, growth factors (e.g., BMP-2), and active constituents of traditional Chinese medicine, enabling the construction of functionally enhanced tissue engineering composites [438]. By providing both biochemical cues and structural support, it facilitates a synergistic effect in bone regeneration. It has also been reported that corn protein-coated bioactive glass-based scaffold loaded with kaempferol can enhance the osteogenic activity of BMSCs through the bioactive properties of kaempferol, thereby accelerating the regeneration of critical-sized bone defects [439].

### 4.4. Organic–Inorganic Composite Scaffolds

Natural bone tissue is a sophisticated composite structure formed through the precise integration of organic components, which impart toughness, and inorganic components, which provide mechanical rigidity. Consequently, an ideal bone scaffold material should overcome the limitations associated with single-phase materials by mimicking the natural composition and hierarchical architecture of bone through the synergistic combination of organic and inorganic phases [440]. The primary advantage of such composite scaffolds lies in their capacity to achieve structural and functional integration. The inorganic phase primarily replicates the mineralized matrix of bone, offering mechanical support, serving as a critical substrate for cell adhesion, and promoting osteogenic differentiation [441]. In contrast, the organic phase constructs a fine three-dimensional porous network that enhances material flexibility, prevents brittle failure, and facilitates the diffusion of nutrients, cells, and biochemical signals [441]. Through precise microscale integration of these two phases, the degradation kinetics of the scaffold can be finely tuned to match the rate of new bone formation, thereby establishing a highly biomimetic microenvironment that effectively supports the repair and regeneration of bone defects [441].

Representatively, poly (lactic-co-glycolic acid) (PLGA)/β-TCP scaffolds have increasingly attracted significant interest in the field of bone tissue engineering [442,443]. These biodegradable scaffolds exhibit excellent biocompatibility, along with favorable porosity and mechanical properties. They facilitate cell adhesion, provide structural support and resistance to shear forces during the early post-implantation phase, and promote the formation of reparative bone tissue, thereby serving as a promising platform for bone repair applications [444,445]. Lai et al. employed PLGA/β-TCP/icariin 3D-printed scaffolds to achieve a synergistic effect between controlled drug release and osteogenic activity [446]. An icaritin-loaded PLGA/TCP scaffold was developed to enhance bone defect repair in steroid-associated osteonecrosis (SAON) [447]. Zhang et al. constructed a PLGA/TCP scaffold co-loaded with icaritin and human fetal mesenchymal stem cells (HFMSCs), which significantly promoted bone regeneration in femoral defects of ovariectomized rats [448]. Their subsequent research demonstrated that, by synergistically combining icaritin with HFMSCs, the PLGA/TCP porous scaffold was endowed with enhanced bioactivity [448]. This created a favorable microenvironment that recruited endogenous MSCs and directed their osteogenic lineage commitment, thereby facilitating osteoporotic bone regeneration [448]. Utilizing low-temperature deposition 3D printing technology, they successfully fabricated PLGA/β-TCP/icaritin composite scaffolds for the treatment of steroid-associated osteonecrosis [449]. The synergistic effects of PLGA’s biodegradability, β-TCP’s osteoconductivity, and icaritin’s bioactivity significantly enhanced osteogenic differentiation and mineralization in bone defect areas, leading to superior bone repair outcomes [111,449,450]. Also, Chen et al. developed a PLGA/β-TCP/icaritin composite scaffold incorporating Ti_3_C_2_T_x_ MXene, which promotes osteogenesis through near-infrared-mediated mild photothermal therapy [111]. To construct a photoresponsive system for bone repair, Gu et al. developed a PLGA/β-TCP scaffold functionalized with PLGA/MXene microspheres loaded with icaritin [451]. This design enables the controlled release of icaritin upon near-infrared irradiation, thereby enhancing bone regeneration [451]. As for other PLGA-based composite scaffolds, Fan’s research team prepared a gelatin-conjugated caffeic acid-coated calcium phosphate/PLGA composite scaffold for the localized and targeted delivery of Tribbles homolog 3 to bone defect sites [452]. This scaffold can direct activate osteogenic differentiation while suppressing adipogenic lineage commitment [452]. Animal studies confirmed the scaffold’s efficacy in significantly enhancing bone regeneration within rat mandibular non-union defects [452]. Furthermore, it effectively inhibited the formation of fat-filled cysts [452]. This combined effect demonstrates a novel therapeutic strategy for bone defect repair that integrates mechanical support with precise signal regulation [452].

Furthermore, Shen et al. constructed a silk fibroin/icariin-loaded mesoporous bioactive glass nanoparticle composite scaffold, which achieves long-term drug loading through its mesoporous structure and enhances the proliferation and osteogenic differentiation of BMSCs [453]. Jing et al. utilized icariin-loaded 45S5 bioactive glass in combination with implanted rat ASCs to promote bone healing and angiogenesis in a rat cranial defect model [454]. The results demonstrated that icariin significantly enhanced the bone healing capacity of 45S5 bioactive glass when co-cultured with rat ASCs [454]. And not coincidentally, based on 45S5 bioactive glass, Reiter et al. developed gelatin-coated 3D sponge-like scaffolds loaded with icariin, which expanded the drug delivery capability through the porous structure and provided morphological flexibility for localized drug delivery [455].

Besides PLGA- and bioactive glass-based composite scaffold, Zhao et al. developed a one-step in situ emulsion polymerization strategy to construct a thiolated icariin-functionalized methacrylic anhydride-modified collagen/hydroxyapatite biomimetic scaffold [456]. This methodology enables the in situ encapsulation of PLGA@icariin microspheres, thereby significantly improving the encapsulation efficiency and sustained-release characteristics of icariin [456]. In vitro and in vivo experimental results demonstrate that the scaffold can modulate the immune microenvironment and activate the vascular-bone regeneration cascade [456]. Moreover, Jin et al. fabricated an icariin-loaded silk fibroin/chitosan/nano-hydroxyapatite composite scaffold [457]. It can promote the repair of critical-sized radial bone defects in rabbits by upregulating osteogenic differentiation-and angiogenesis-related genes and proteins [457]. Similar study of Xie et al. reported. an icariin-loaded hydroxyapatite/alginate porous composite scaffold capable of sustained icariin release to enhance bone regeneration by mimicking the bone mineralization microenvironment [458]. In addition, researchers developed a 3D-printed polycaprolactone scaffold that incorporates both strontium-substituted mesoporous bioactive glass nanoparticles and icariin [459]. This composite system enables the sustained release of Sr^2+^ ions and icariin [459]. Together, these agents promote osteoporotic bone regeneration through a dual mechanism: enhancing osteoblast proliferation and differentiation while simultaneously suppressing osteoclastogenesis [459]. Yang et al. developed a functionalized decellularized mushroom stem scaffold composed of naturally aligned channel structures, zinc ion/curcumin metal–organic frameworks, hydroxyapatite minerals, and icariin [460]. This scaffold can control bacterial infection, promote the migration of BMSCs, and further accelerate M2 macrophage polarization and osteogenic differentiation of BMSCs [460].

### 4.5. Nanoparticles

Nanoparticle-based drug delivery systems exploit size-dependent circulatory properties (10–200 nm) to achieve passive targeting, thereby enabling extravasation into osseous defect regions. Through stimulus-responsive designs, such as pH-and temperature-sensitive mechanisms, these systems allow for controlled drug release within defined microenvironments, including acidic pathological niches. Moreover, the intrinsic bone-targeting affinity of nanomaterials contributes to reduced immunogenicity and cytotoxicity. There is no doubt that nanoparticles represent one of the most effective delivery systems for bioactive components derived from Chinese herbal medicines.

As for metal or metal oxide nanoparticles, Zhang et al. synthesized dopamine-poly (ethylene glycol)-coated porous hollow iron oxide nanoparticles through thermal decomposition and controlled oxidation techniques, and encapsulated icariin within their porous hollow structures [461]. In vitro studies demonstrated that the nanoparticle system can promote osteogenic differentiation [461]. And in vivo studies revealed that under the application of an external magnetic field, the nanoparticles can accumulate at the fracture site, thereby accelerating fracture healing through the localized release of icariin. Similarly, Wang et al. proposed the incorporation of biocompatible iron oxide nanoparticles and icariin into a nano-scaffold fabricated via coaxial electrospinning technology, which significantly enhanced the osteogenic performance of the scaffolds in vivo [462]. Moreover, Yang et al. successfully constructed a nanohybrid system by combining chlorogenic acid with gold nanorods [463]. These nanohybrids are capable of inducing apoptosis and inhibiting the proliferation of osteosarcoma cells [463]. Furthermore, under controlled and mild near-infrared irradiation, the nanohybrids stimulate osteogenic differentiation by upregulating the expression of Hsp47 and Hsp70 genes and promoting the production of heat shock proteins [463]. Furthermore, Yuan et al. developed a dual-controlled release system for drugs and bioactive ions by loading hydrophobic icariin into MgO/MgCO_3_ nanoparticles and subsequently encapsulating the nanoparticles at a fixed ratio (20%) within biodegradable PLGA microspheres, thereby achieving sustained release of both magnesium ions and icariin [464]. This system not only effectively promotes new bone formation but also significantly enhances osteogenic induction capacity [464]. However, metal and metal oxide nanocarrier systems continue to face significant challenges in clinical applications. The primary concern is their biocompatibility and potential for long-term toxicity [465]. Certain metal ions or nanoparticles may induce oxidative stress, provoke inflammatory responses, and lead to organ accumulation, while their metabolic pathways and long-term biodistribution in the body remain poorly understood [465]. Secondly, the inherent chemical properties of these materials can compromise stability, resulting in premature drug leakage, particle aggregation, or degradation under physiological conditions—factors that impair targeting accuracy and therapeutic effectiveness [465]. Finally, there is a risk of off-target effects and immunogenicity, as nanoparticles may be recognized and cleared by the reticuloendothelial system or interact non-specifically with non-target tissues, potentially causing unintended adverse reactions [465]. Collectively, these limitations hinder the clinical translation of such nanocarriers.

For these reasons, various organic nanoparticles and organic/inorganic composite nanoparticles have been explored as delivery vehicles for bioactive components derived from Chinese herbal medicines. Representative examples of successful applications are as follows: Yan et al. designed a novel therapeutic strategy for osteoporosis based on erythrocyte membrane-coated nanoparticles [466]. To enhance biocompatibility, icariin was encapsulated within a poly(D,L-lactide) core, and the surface was functionalized with erythrocyte membranes [466]. Targeting specificity was further improved by synthesizing alendronate-modified 3-phosphoethanolamine-N-methoxy(polyethyleneglycol)-carboxyl bone-targeting polymers, which were incorporated into the membrane coating [466]. This multifunctional nanoparticle system demonstrated significant synergistic effects, promoting both angiogenesis and osteogenesis in vitro [466]. Luo et al. constructed a nanocargo system by loading icariin onto polydopamine-modified covalent organic frameworks [467]. The resulting nanocomplex was shown to synergistically ameliorate the local inflammatory microenvironment via immunomodulation and enhance bone defect repair by promoting osteogenic differentiation [467]. There have also been several attempts to design delivery systems for other bioactive components derived from *Epimedium*. For instance, Feng et al. developed a pH-responsive HA-modified anti-inflammatory catechin-based metal–organic framework system [468]. It can significantly delay the progression of osteoarthritis by inhibiting Col II degradation and suppressing IL-1β-stimulated inflammatory mediator production [468]. Also, Li et al. prepared a polyvinylpyrrolidone-modified PLGA-based drug delivery system, which achieves long-term controlled release through co-loading of chlorogenic acid [469]. This system not only efficiently scavenges ROS in the periodontal microenvironment but also disrupts the pathological cycle of inflammation-oxidative stress by suppressing the overexpression of pro-inflammatory cytokines [469]. The scaffold can significantly inhibit alveolar bone resorption and synergistically block the progression of periodontitis through a triple mechanism involving anti-oxidant activity, anti-inflammatory effects, and bone-protective functions [469]. Tactfully, a novel therapeutic strategy based on calcium-chlorogenic acid nanoparticles was developed [470]. The nanoparticles can promote the differentiation of BMSCs into osteoblasts and induce macrophage polarization toward the M2 phenotype [470]. This dual regulatory effect significantly enhances bone repair [470]. Even more interestingly, a research team fabricated a novel supramolecular nano-agonist through the self-assembly of berberine and chlorogenic acid [471]. When the assembly ratio of chlorogenic acid/berberine changes from 1:1 to 1:4, the nano-agonist can passively target osteoporotic bone tissue after prolonged systemic circulation, modulate the Wnt signaling pathway, and thereby regulate osteogenic differentiation [471]. Moreover, a co-nanoparticle formulation of luteolin and palmitoylethanolamide has been shown to significantly reduce pro-inflammatory cytokines (e.g., IL-6, TNF-α) and oxidative stress markers (e.g., nitrotyrosine, malondialdehyde), thereby alleviating joint erosion [472]. Lee et al. formulated kaempferol-loaded gelatin nanoparticles coated with HA (HA-KM GNPs) [473]. The administration of HA-KM GNPs in the anterior cruciate ligament transection rat models markedly attenuated the progression of knee osteoarthritis (KOA) [473]. The treatment effectively reduced subchondral sclerosis, alleviated inflammation, inhibited extracellular matrix degradation, and restored cartilage thickness, thereby lessening overall KOA severity [473]. Additionally, a novel nano complex composed of CTS, chondroitin sulfate, and daidzein was prepared for the treatment of glucocorticoid-induced osteoporosis through targeted delivery and sustained release of daidzein [474]. This nanocomplex enhanced osteoblast formation by upregulating ALP and OPG expression, while reducing osteoclast activity [474].

Polymer micelles are nanoscale core–shell structures formed through the self-assembly of amphiphilic block copolymers in selective solvents. The hydrophobic core serves as an ideal reservoir for poorly water-soluble drugs, thereby significantly improving drug solubility and stability [475]. And the hydrophilic shell confers excellent aqueous dispersibility, prolonged systemic circulation, and passive targeting capability to the micelles [475]. Furthermore, by surface functionalization, these nanocarriers can achieve efficient and intelligent delivery to specific cells or tissues, demonstrating considerable potential for applications in nanomedicine [475]. It has been reported that polymeric micelles loaded with icaritin can effectively treat various diseases by enhancing its solubility, stability, and bioavailability [476,477]. Sun et al. demonstrated that alendronate-functionalized polymer micelles for targeted delivery of icaritin to osteoporotic bone effectively alleviated osteoporosis in rat models and partially restored bone microarchitecture [478]. Tang et al. employed an innovative acid-base conversion method to prepare spherical polymeric micelles with high drug loading capacity, using Soluplus^®^ and Poloxamer 407 as matrix materials for icaritin encapsulation, thereby enhancing both the drug loading capacity and oral bioavailability of icaritin [477]. The fatty acids present in sheep oil (SO) possess long hydrocarbon chains and surfactant-like properties, enabling them to self-assemble with bile salts in vivo to form nanomicelles [479,480]. These nanomicelles can serve as drug delivery carriers and facilitate drug absorption [481,482]. Jun et al. exploited this characteristic to develop an icaritin-SO-deoxycholate self-assembled nanomicelle system, which reduced the particle size of icaritin, thereby enhancing its absorption, improving bioavailability, and further augmenting its anti-osteoporotic efficacy [483]. Moreover, Akshay et al. synthesized mPEG-b-PCL block copolymers through ring-opening polymerization of ε-caprolactone [484]. And they conjugated the hydrophilic compound caffeic acid to mPEG-b-PCL micelles via Steglich esterification and incorporated the therapeutic agent 9-aminoacridine [484]. These nanomicelles were shown to alleviate arthritic symptoms by reducing inflammation, joint damage, bone erosion, and swelling [484].

Both polymer micelles and liposomes are nanocarriers for drug delivery formed through the self-assembly of amphiphilic molecules. However, polymer micelles typically accommodate only hydrophobic drugs, whereas liposomes are capable of encapsulating both hydrophilic and hydrophobic therapeutic agents simultaneously [485,486]. The application of icaritin-loaded liposomes in the treatment of bone-related diseases has also demonstrated promising therapeutic outcomes [487]. The bioavailability of icaritin in this formulation was approximately 28-fold higher than that of free icaritin [487]. Moreover, Chen et al. developed a novel bone-targeted delivery system, i.e., Asp8-liposome-icaritin [487]. Asp8 enhances the specific targeting of the *Epimedium*-derived molecule icaritin, which is encapsulated within liposomes, to bone remodeling sites [487]. This system can promote bone formation by regulating the differentiation of BMSCs into osteoblasts, upregulating osteoblast function, and inhibiting osteoclast activity [487]. Furthermore, it effectively prevents osteonecrosis by maintaining a high local concentration of icaritin on the bone surface [487].

### 4.6. Extracellular Vesicles

Extracellular vesicles (EVs) have demonstrated significant potential in the field of controlled drug release, owing to their inherent biological activity and intelligent delivery capabilities [487,488]. As naturally occurring nanoscale carriers, they exhibit excellent biocompatibility, low immunogenicity, and the ability to cross physiological barriers, enabling effective protection of therapeutic cargo and targeted delivery [489]. In the context of controlled release, the lipid bilayer of extracellular vesicles can function as a “switch”, undergoing rupture in response to specific physicochemical stimuli in the pathological microenvironment (such as acidic conditions or the presence of specific enzymes), thus facilitating precise and stimulus-triggered drug release [489]. Furthermore, through surface engineering or hybridization with synthetic materials, their release kinetics can be precisely programmed [489].

In recent years, numerous researchers have sought to engineer EVs as carriers for the bioactive components of *Epimedium* in the context of bone tissue engineering. Yu et al. isolated small EVs (sEVs) from bovine milk and subsequently prepared sEV@icariin complexes [490]. These complexes promote osteogenesis by facilitating the binding of the signal transducer and activator of transcription 5A to the gap junction protein alpha 1 promoter, exhibiting enhanced efficacy in bone repair both in vitro and in vivo [490]. Also, Yan et al. developed an adipose-derived stem cell exosome-based system for icariin delivery [491]. Experimental studies demonstrated that the nanomaterial inhibits glycolysis, thereby modulating macrophage polarization from the M1 to M2 phenotype and alleviating rheumatoid arthritis [491]. Although EV-based carriers have shown considerable potential in bone tissue engineering, the targeting and homing capability of natural vesicles remains limited, and their retention time at the lesion sites is difficult to regulate. Consequently, they are often required to be combined with scaffold materials to enhance therapeutic efficacy. Inspired by mussel-inspired chemistry, Zeng et al. also fabricated a multifunctional hydrogel system capable of co-delivering BMSC-derived exosomes and icariin, achieving a synergistic therapeutic effect [492]. More importantly, due to its prolonged retention in vivo, the system enhances the protective effect on cartilage [492]. Meanwhile, in the osteoarthritis model, the co-delivery hydrogel system effectively alleviated cartilage atrophy and maintained appropriate cartilage thickness [492].

### 4.7. Bioactive Coatings for Implants

Currently, many soft and hard tissue implant materials used in clinical practice exhibit weak interactions with host tissues due to surface biological inertness [3,4,94,493,494]. The construction of bioactive coatings is widely regarded as a simple, cost-effective, and efficient approach to enhance the biological performance of implant material surfaces. Among them, the sustained-release coating of bioactive substances derived from Chinese herbal medicines, enabled by precise compositional regulation and endowed with programmable functionalities, effectively meets the personalized functional demands of implant surfaces and is currently recognized as a highly promising surface modification strategy [495,496]. First of all, PEEK as well as Ti and its alloys, which are widely used in clinical practice, are taken as examples. He et al. addressed critical challenges in clinical bone implants, including large bone defects and associated infections, by integrating a chlorogenic acid/grafted peptide hydrogel onto sulfonated PEEK surfaces [497]. A study revealed that the surface of a Ti implant coated with quercitrin can effectively reduce the expression of osteoclast functional markers, such as CTSK, H+ATPase, and MMP9, both in vivo and in vitro [498]. This effect contributes to the maintenance of marginal bone levels and enhances long-term implant stability [498]. The functionalization of the titanium surface with quercetin and paclitaxel was shown to confer excellent osteogenic, anti-inflammatory, and anti-fibrotic properties on the implant, thereby promoting rapid osseointegration between the implant and host bone [498,499]. Yang et al. developed novel caffeic acid nanospheres and deferoxamine-based films coated on Ti implant to modulate ferroptosis. The intelligent Ti implant was capable of alleviating iron overload and oxidative stress in postmenopausal osteoporosis and enhancing osseointegration [500]. Moreover, the porous structure of quercetin-functionalized Ti6Al4V orthopedic implant has been demonstrated to preserve the mechanical integrity of the implant, including its Young’s modulus, while exhibiting favorable cytocompatibility [501]. This design also supports the proliferation and differentiation of bone cells, reduces bacterial adhesion and viability, and simultaneously enhances osseointegration and prevents implant-associated infections [501]. Giacomo et al. employed hexamethylenediamine to modify self-oxidized caffeic acid, endowing it with specific film-forming capabilities [502]. The approach enabled the formation of thin, uniform, and stable coatings on polished and chemically pre-treated Ti6Al4V alloys [502]. These coatings exhibited high cytocompatibility with stem cells involved in tissue regeneration and demonstrated effective scavenging activity, thereby protecting cells from apoptosis induced by toxic reactive species in pro-inflammatory microenvironments [502]. As for other types of implants, a thiolated icariin derivative (icariin-SH), characterized by favorable cytocompatibility and biological activity, was immobilized onto a BCP scaffold through intermolecular disulfide bond formation among icariin-SH molecules, forming a thin-film coating (icariin-SH/BCP) [503]. The icariin-SH/BCP scaffold can promote the proliferation, migration, and osteogenic differentiation of osteoporotic rat BMSCs, while also upregulating the expression of angiogenic genes in HUVECs in vitro [503]. In addition, the OVX rat femoral condyle defect model demonstrated that the icariin-SH/BCP scaffold significantly enhanced bone regeneration in osteoporotic animals compared to the conventional BCP scaffold [503].

**Table 2:** Bone tissue engineering strategies for delivering core bioactive components of *Epimedium* in the treatment of bone defects.

Carriers	Active Constituents	Applications	Bone Functions	References
Hydrogel	Icariin	Icariin–zinc–aluminum layered double hydroxide nanosheets-GA-hydroxybutyl CTS hydrogel	Promoted periodontal tissue	[401]
		Poly(N-vinyl caprolactam)@icariin nanoparticles encapsulated in hydroxyethyl methacrylate-functionalized thermosensitive hydrogels	Treated critical bone and cartilage injuries	[402]
		Icariin-loaded 3D-Ti/CTS hydrogel	Promoted osteogenesis and osseointegration	[403]
		Icariin-loaded HA/CTS/Ti composite hydrogel	Improved osteoblast response and osseointegration	[404]
		Icariin-loaded CTS/hydroxyapatite composite hydrogel	Enhanced bone repair and regeneration	[405]
		Icariin/CTS/β-glycerophosphate hydrogel	Controlled infection and repaired bone tissue	[406]
		Icariin-conditioned serum combined with thiolated CTS	Attenuated cartilage injury in rabbit knees with osteochondral defect	[407]
		Self-assembled injectable icariin@Ti_3_C_2_T_x_ /DOX sodium alginate hydrogel	Significantly improved osteosarcoma’s therapeutic efficacy while offered a novel approach to mitigate the osteogenic inhibitory effects of DOX	[408]
	Icaritin	Injectable icaritin@Gelatin/Ac-β-CD hydrogel	Speeded up the in situ bone regeneration in a SAON animal model	[13]
		Icaritin/DOX-loaded PF127/PF68 hydrogel	Contributed to bone regeneration, offering significant potential for enhancing OS treatment outcomes and promoting healing of affected bone tissue	[409]
	Ginkgetin	Ginkgetin/rapamycin-loaded MMP13-responsive nanogel	Suppressed inflammation and rescued the blocked autophagic flux without inducing cell death, demonstrating a notable therapeutic effect in Intervertebral disc degeneration (IVDD)	[412]
	Magnoflorine	A magnoflorine-loaded HA hydrogel	Enhanced subchondral bone regeneration and prevented osteoarthritis progression following anterior cruciate ligament rupture	[413,414]
	β-sitosterol	Gelatin/CTS/HA@Diatoms and β-sitosterol	Promoted osteoblast adhesion, proliferation, and mineralization	[415]
	Caffeic acid	Nanoclay-reinforced caffeic acid-modified CTS hydrogel	Regulated the Wnt/β-catenin pathway, along with the addition of osteoinductive signals, resulting in bone regeneration in a nonhealing cranial defect	[416]
		Caffeic acid/citric acid-functionalized poly (xylitol succinate)@magnesium oxide injectable hydrogel	Exhibited antagonistic properties against TLR4 and Myeloid differentiation factor 2 complex and reduced inflammatory cytokines levels and maintains subgingival microbial homeostasis	[417]
	Chlorogenic acid	Chlorogenic acid-loaded calcium phosphate/CTS porous nanogel	Degraded the biofilm and also be used in the restoration of tooth defects	[418]
	Neochlorogenic acid	A poly-methylacrylamide hydrogel encapsulating neochlorogenic acid and BMSCs	Mitigated oxidative stress and apoptosis, enhancing BMSC survival for IVDD repair	[419]
Organic scaffold	Icariin	Icariin-delivering porous poly (3-hydroxybutyric acid) scaffold	Regenerative repair of femoral head necrosis in rabbits	[420]
		Icariin-loaded 3D-printed PCL scaffolds	Improved the clinical outcome of alveolar cleft reconstruction	[421]
		Gradient gelatin methacryloyl/polyethylene glycol diacrylate matrix scaffold encapsulating PolyApt@icariin and PolyApt@EGCG	Achieved perfect structural and functional regeneration in vivo	[422]
		Icariin-derived ECM-ornamented SIS scaffold	Exhibited superior osteoconductivity, enhanced cell adhesion, spreading, survival, and proliferation, while not compromising osteoinductivity	[423]
Inorganic scaffold	Icariside II	Icariside II-loaded CPC scaffold	Promoted bone regeneration by enhancing the proliferation and survival of BMSCs in vivo	[432]
	Luteolin	Luteolin-loaded biocomposites containing tantalum and polyimide	Facilitated osteogenic differentiation and bone bonding	[433]
	Icariin	Sr-doped BCP/icariin	Enhanced osteogenesis by activating the Wnt/β-catenin and BMP-2/Smad signaling pathways and attenuated osteoclast activity via suppression of the NF-κB/p38 MAPK pathway	[434]
	Icaritin	Icaritin-functionalized β-TCP composite scaffold	Regenerative repair of femoral head necrosis in rabbits	[436]
Organic- inorganic composite scaffold	Icariin	PLGA/TCP/icariin 3D-printed scaffold	Promoted bone formation	[446]
		A silk fibroin/icariin-loaded mesoporous bioactive glass nanoparticle composite scaffold	Enhanced the proliferation and osteogenic differentiation of BMSCs	[453]
		Icariin-loaded 45S5 bioactive glass/implanted rat ASCs	Enhanced the bone healing capacity	[454]
		Icariin-loaded gelatin-coated 3D sponge-like scaffold/45S5 bioactive glass	Expanded icariin delivery capability and provided morphological flexibility for localized drug delivery	[455]
		Poly(lactide-co-glycolide)@icariin microspheres encapsulated in thiolated icariin-functionalized methacrylic anhydride-modified collagen/hydroxyapatite biomimetic scaffold	Modulated the immune microenvironment and activated the vascular-bone regeneration cascade	[456]
		An icariin-loaded silk fibroin/chitosan/nano-hydroxyapatite composite scaffold	Promoted the repair of critical-sized radial bone defects in rabbits	[457]
		Icariin-loaded hydroxyapatite/alginate porous composite scaffold	Enhanced bone regeneration	[458]
		Icariin/strontium-substituted mesoporous bioactive glass nanoparticles loaded 3D-printed PCL scaffold	Enhanced osteoblast proliferation and differentiation	[459]
		Icariin/zinc ion/curcumin metal–organic frameworks/hydroxyapatite@Functionalized decellularized mushroom stem scaffold with aligned channel structures	Controlled bacterial infection and promoted bone marrow mesenchymal stem cell migration and accelerated M2 macrophage polarization and osteogenic differentiation	[460]
	Icaritin	An icaritin-loaded PLGA/TCP scaffold	Enhanced bone defect repair in SAON	[447]
		A PLGA/TCP scaffold loaded with icaritin and HFMSCs	Facilitated osteoporotic bone regeneration	[448]
		PLGA/β-TCP/icaritin composite scaffolds	Enhanced osteogenic differentiation and mineralization in bone defect areas, leading to superior bone repair outcomes	[449,450]
		A PLGA/β-TCP/icaritin composite scaffold incorporating Ti_3_C_2_T_x_ MXene	Promoted osteogenesis through near-infrared-mediated mild photothermal therapy	[111]
		Icaritin-loaded PLGA/MXene microspheres on PLGA/β-TCP scaffold	Enhanced bone regeneration	[451]
	Caffeic acid	A novel gelatin-conjugated caffeic acid-coated apatite/PLGA composite scaffold	Enhanced bone regeneration and inhibited the formation of fat-filled cysts	[452]
Nanoparticles	Icariin	Icariin@dopamine-poly (ethylene glycol)-coated porous hollow iron oxide	Promoted osteogenic differentiation and accelerated fracture healing	[461]
		Icariin/iron oxide nanoparticles@ coaxial electrospun nano-scaffold	Enhances the osteogenic performance of the scaffolds in vivo	[462]
		Icariin@MgO/MgCO_3_/PLGA microspheres	Promoted new bone formation and enhanced osteogenic induction capacity	[464]
		Icariin @ alendronate-modified 3-phosphoethanolamine-N-methoxy(polyethyleneglycol)-carboxyl erythrocyte membrane-coated poly(D,L-lactide) nanoparticles	Promoted both angiogenesis and osteogenesis in vitro	[466]
		Icariin@polydopamine-modified covalent organic frameworks	Ameliorated local inflammatory microenvironment and promoted osteogenic differentiation.	[467]
	Icaritin	Icaritin@Soluplus^®^/Poloxamer 407 micelles	Enhanced both the drug loading capacity and oral bioavailability of icaritin	[477]
		Icaritin@SO-deoxycholate self-assembled nanomicelle	Enhanced the ability to resist osteoporosis.	[483]
		Icaritin@Asp8-liposomes	Promoted bone formation and upregulated osteoblast function and inhibited osteoclast activity and prevents osteonecrosis	[487]
	Caffeic acid	Caffeic acid/9-aminoacridine@mPEG-b-PCL	Inhibited articular cartilage deterioration and reduced disease severity in experimental inflammatory arthritis	[484]
	Chlorogenic acid	Chlorogenic acid@Gold nanorods (AuNRs)	Inhibited osteosarcoma proliferation and stimulated osteogenic differentiation	[463]
		Chlorogenic acid@polyvinylpyrrolidone-modified PLGA-based drug delivery system	Scavenged ROS and disrupted the pathological cycle of inflammation-oxidative stress and inhibited alveolar bone resorption	[469]
		Calcium-chlorogenic acid nanoparticles	Promoted the differentiation of BMSCs into osteoblasts and induced macrophage polarization toward the M2 phenotype	[470]
		Berberine/chlorogenic acid supramolecular nano-agonist	Regulated osteogenic differentiation	[471]
	Catechin	Catechin@pH-responsive HA-modified metal–organic framework	Delayed the progression of osteoarthritis	[468]
	Luteolin	Luteolin/palmitoylethanolamide co-nanoparticles	Alleviated joint erosion	[472]
	Kaempferol	Kaempferol@HA-coated gelatin nanoparticles	Attenuated the progression of KOA	[473]
	Daidzein	Daidzein@CTS/chondroitin sulfate nano complex	Enhanced osteoblast formation and reduced osteoclast activity	[474]
Extracellular vesicles	Icariin	Bovine milk derived sEV@icariin	Treated bone defect and improved the osteogenic activity of icariin	[490]
		An adipose-derived stem cell exosome@icariin	Anti-rheumatoid arthritis and inhibition of macrophage function	[491]
		A hydrogel system capable of co-delivering BMSC-derived exosomes and icariin	Alleviated cartilage atrophy and maintained appropriate cartilage thickness	[492]
Bioactive coatings for implants	Icariin	Icariin-SH/BCP	Intended for the repair of bone defects in patients	[503]
	Quercitrin	Ti implant coated with Quercitrin	Reduced the expression of osteoclast functional markers, such as CTSK, H+ATPase, and MMP9, both in vivo and in vitro	[498]
		Quercitrin-functionalized Ti6Al4V orthopedic implant	Promoted the bone integration of orthopedic implants and prevented infections	[501]
	Chlorogenic acid	Chlorogenic acid/grafted peptide hydrogel-modified sulfonated PEEK surfaces	Promoted bone tissue repair and eliminated bacteria	[497]
	Caffeic Acid	Caffeic acid nanospheres and deferoxamine-based films@titanium implant surface	Alleviated iron overload and oxidative stress in postmenopausal osteoporosis and enhanced osseointegration	[500]
		Hexamethylenediamine-modified self-oxidized caffeic acid coating@Ti6Al4V alloy surface	Good cell compatibility and high ability to eliminate reactive oxygen species	[502]

## 5. Prospects

Although *Epimedium* has been widely used in clinical medicine for a long time and has undergone extensive scientific research, several challenges remain regarding its application in the field of bone tissue engineering. Firstly, studies on the functional and molecular mechanisms of *Epimedium* in regulating bone metabolism remain insufficiently systematic and comprehensive. For example, the cellular metabolic pathways of icaritin, as well as the intestinal metabolism and tissue distribution of *Epimedium* in bone, have not been adequately investigated. Secondly, insufficient controllability of active ingredients: The composition of *Epimedium* extract is highly complex, and the concentration of its bioactive components is significantly influenced by factors such as geographical origin and extraction methodology, posing challenges to the standardization of its application in scaffold materials or bioinks. Thirdly, poor compatibility of delivery systems: Current delivery carriers exhibit limited sustained-release capacity for active substances, which fails to meet the dynamic demands of bone regeneration. Additionally, the precision of local microenvironment modulation remains inadequate. Fourthly, unclear cross-scale action mechanism: The multi-level mechanisms underlying the transition from molecular effects to tissue regeneration have not yet been fully elucidated, thereby hindering the synergistic design of materials, cells, and host responses. Fifthly, barriers to clinical translation: discrepancies between animal models and the human physiological microenvironment often result in inaccurate efficacy predictions. Furthermore, a robust quality control system for large-scale production is currently lacking.

The following strategic approaches are proposed to effectively overcome the aforementioned challenges. Firstly, researchers are encouraged to integrate single-cell RNA sequencing and spatial transcriptomics to map the spatiotemporal tissue distribution and specific cellular targets of *Epimedium* bioactive constituents, thereby revealing the high-resolution molecular regulatory mechanisms of bone metabolism. Secondly, develop smart systems responsive to stimuli: engineer hydrogels or nanocarriers sensitive to the microenvironment capable of release upon demand (e.g., triggered by pH, reactive oxygen species, or specific enzymes) to achieve precise spatiotemporal modulation during dynamic bone regeneration. Thirdly, biomimetic structural strategies: utilize 3D and 4D bioprinting technology to fabricate dynamic, morphologically adaptable scaffolds that not only mimic the topological architecture of the native bone extracellular matrix but also adapt spatiotemporally to progressive tissue remodeling, thereby enhancing cell recruitment and directed differentiation. Fourthly, AI-driven multi-omics optimization: leverage artificial intelligence algorithms and machine learning to integrate complex metabolomic and spatial omics datasets, constructing a quantitative relationship model among components, structures, and functions to precisely guide the standardization and optimization of protocols. Fifthly, innovative preclinical evaluation methodologies: develop microfluidic organoid platforms and humanized animal models to enable high-throughput, real-time monitoring, effectively bridging interspecies discrepancies and improving the predictive accuracy for clinical translation.

## Figures and Tables

**Figure 1:**
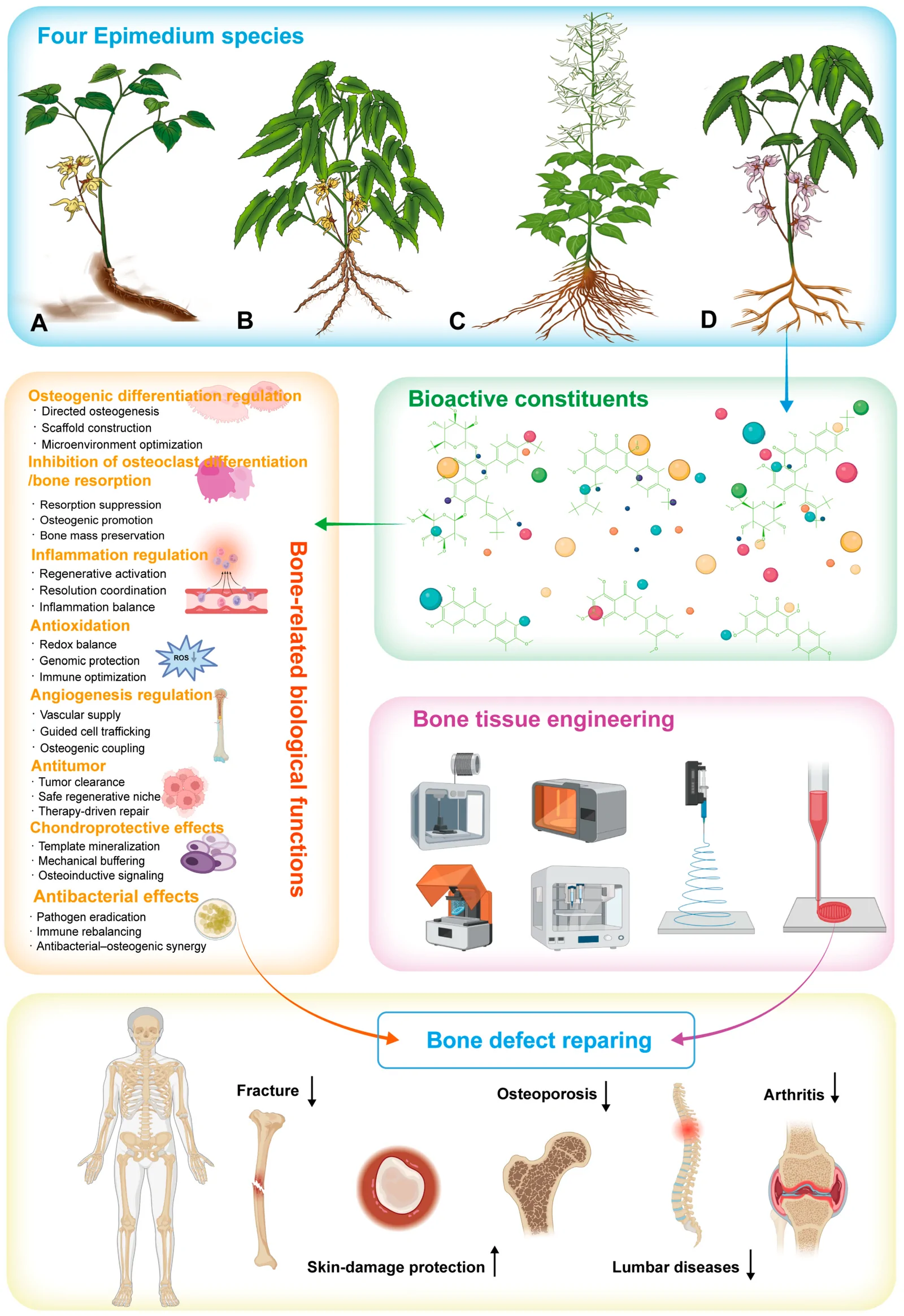
Schematic of “Traditional Medicinal Herb-to-Bone Tissue Engineering-to-Bone Defect Repair”: Integration of bioactive constituents from four *Epimedium* species: (**A**) *Epimedium koreanum* Nakai., (**B**) *Epimedium sagittatum* (Sieb. et Zucc.) Maxim., (**C**) *Epimedium brevicornum* Maxim., and (**D**) *Epimedium sutchuenense* Franch. and their modulation of key biological mechanisms, including osteogenic differentiation, inhibition of osteoclast differentiation and bone resorption, inflammation regulation, antioxidant effects, angiogenesis regulation, antitumor effects, chondroprotective effects, and antibacterial effects, in combination with advanced bone tissue engineering strategies. These functionalized constructs are specifically designed to meet clinical demands, enabling effective repair of bone defects and the treatment of diverse pathologies, including fractures, osteoporosis, and arthritis. The circle colors distinguish different *Epimedium*-derived bioactive constituents, while the circle sizes represent their molecular weights. Arrows show the flow from the four *Epimedium* species to these constituents (blue), from the constituents to their biological effects (green), and from these effects (orange) or tissue-engineering approaches (magenta) to bone defect repair. Black arrows indicate whether the labeled outcomes decrease (↓) or increase (↑).

**Figure 2:**
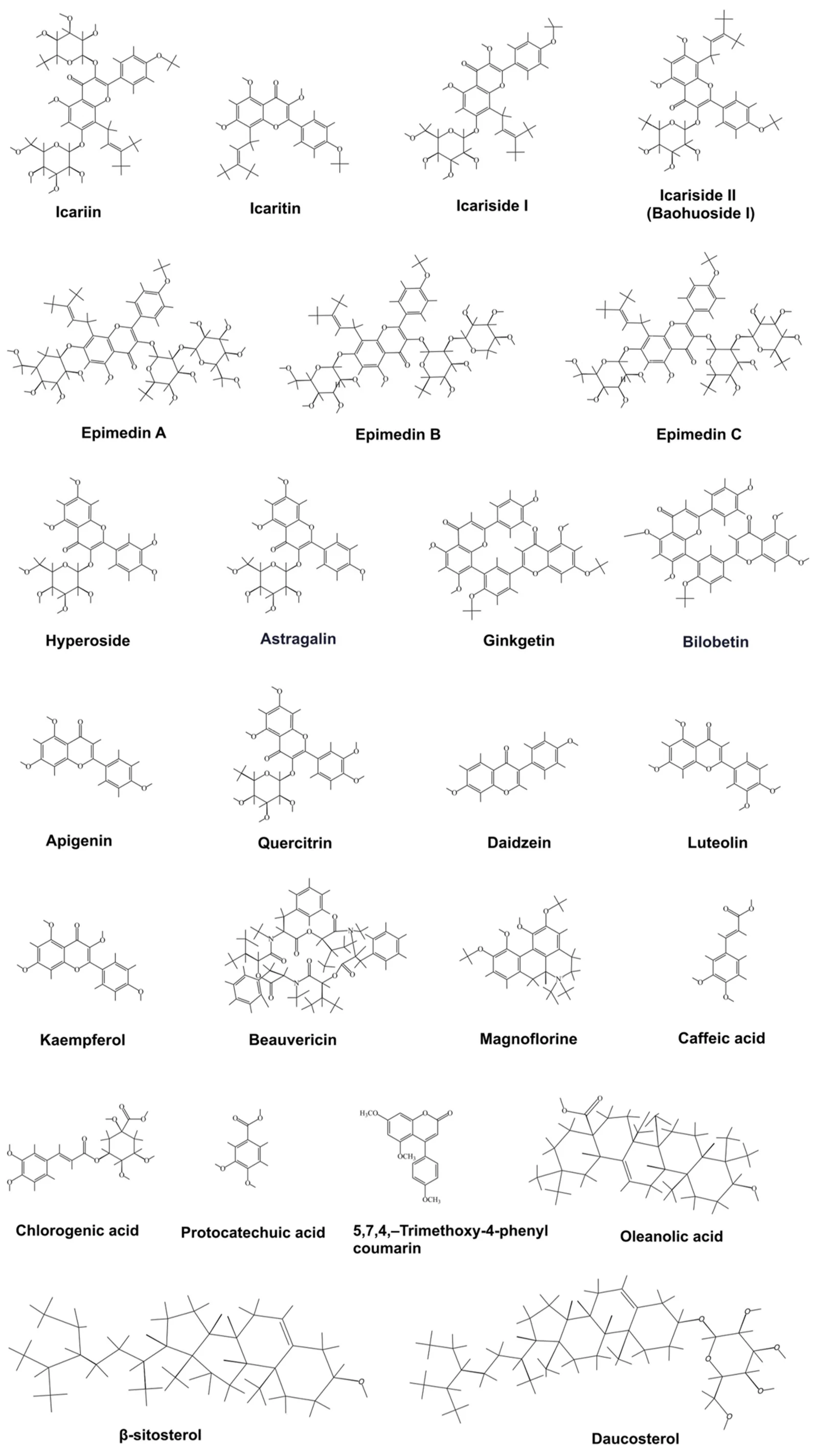
Structural formulae of active constituents of *Epimedium*.

**Figure 3:**
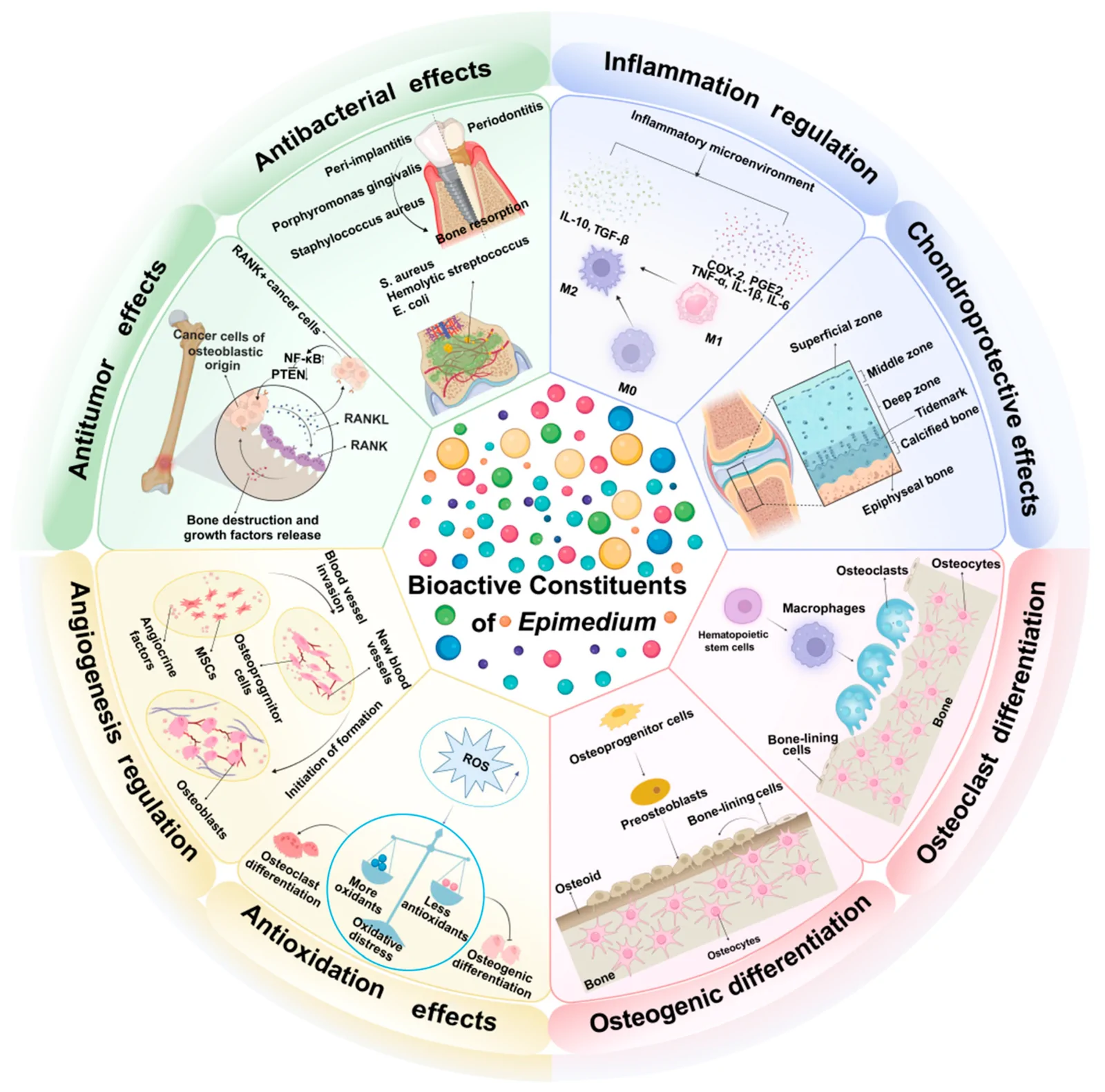
Key molecular mechanisms underlying the bioactive constituents derived from *Epimedium* in relation to bone defect repair and associated biological functions. Several interconnected mechanisms contribute to bone defect repair progression: (1) Osteogenic differentiation regulation: Bioactive constituents promote the differentiation of osteoprogenitor cells into preosteoblasts and mature osteoblasts through activation of osteogenesis-related signaling pathways and transcription factors, leading to osteoid secretion and subsequent initiation of bone matrix mineralization. (2) Inhibition of osteoclast differentiation and bone resorption: Bioactive constituents inhibit osteoclast differentiation by modulating specific signals (e.g., RANKL, HIF-1α, etc.) and signaling pathways (e.g., MAPK, NF-κB, etc.), thereby maintaining the balance between osteogenesis and osteoclastogenesis and promoting bone defect repair. (3) Inflammatory modulation: Bioactive constituents modulate macrophage activity, including M0, M1, and M2 phenotypes, thus suppressing the release of pro-inflammatory mediators (e.g., TNF-α, IL-1β, IL-6, COX-2, PGE2), while promoting anti-inflammatory responses mediated by factors like IL-10 and TGF-β. (4) Antioxidant activity: Bioactive constituents attenuate intracellular reactive oxygen species accumulation and mitigate oxidative stress-induced damage, thereby preserving the regenerative capacity of osteogenic and endothelial cells. (5) Angiogenesis regulation: In regenerating tissues, constituents of *Epimedium* promote endothelial cell migration and sprouting, leading to neovascularization, which subsequently supports the generation and functional development of osteoblasts. (6) Antitumor effects: Bioactive constituents modulate osteolytic signaling pathways between tumor cells and bone-resorbing cells, thereby mitigating tumor-induced bone destruction. (7) Chondroprotective effects: Bioactive constituents contribute to the mitigation of cartilage degeneration, thereby promoting joint stability and facilitating coordinated bone–cartilage repair. (8) Antibacterial effects: Bioactive constituents inhibit the growth of bone-related pathogens, such as *Staphylococcus aureus*, *Streptococcus pyogenes*, and *Escherichia coli*, and mitigate infection-induced inflammatory responses, thereby preventing secondary bone defects. The circle colors distinguish different *Epimedium*-derived bioactive constituents, whereas the circle sizes indicate their relative molecular weights. Arrowheads indicate the direction of a biological process and denote activation, promotion, secretion, or release, as appropriate, whereas blunt-ended lines denote inhibition.

**Figure 4:**
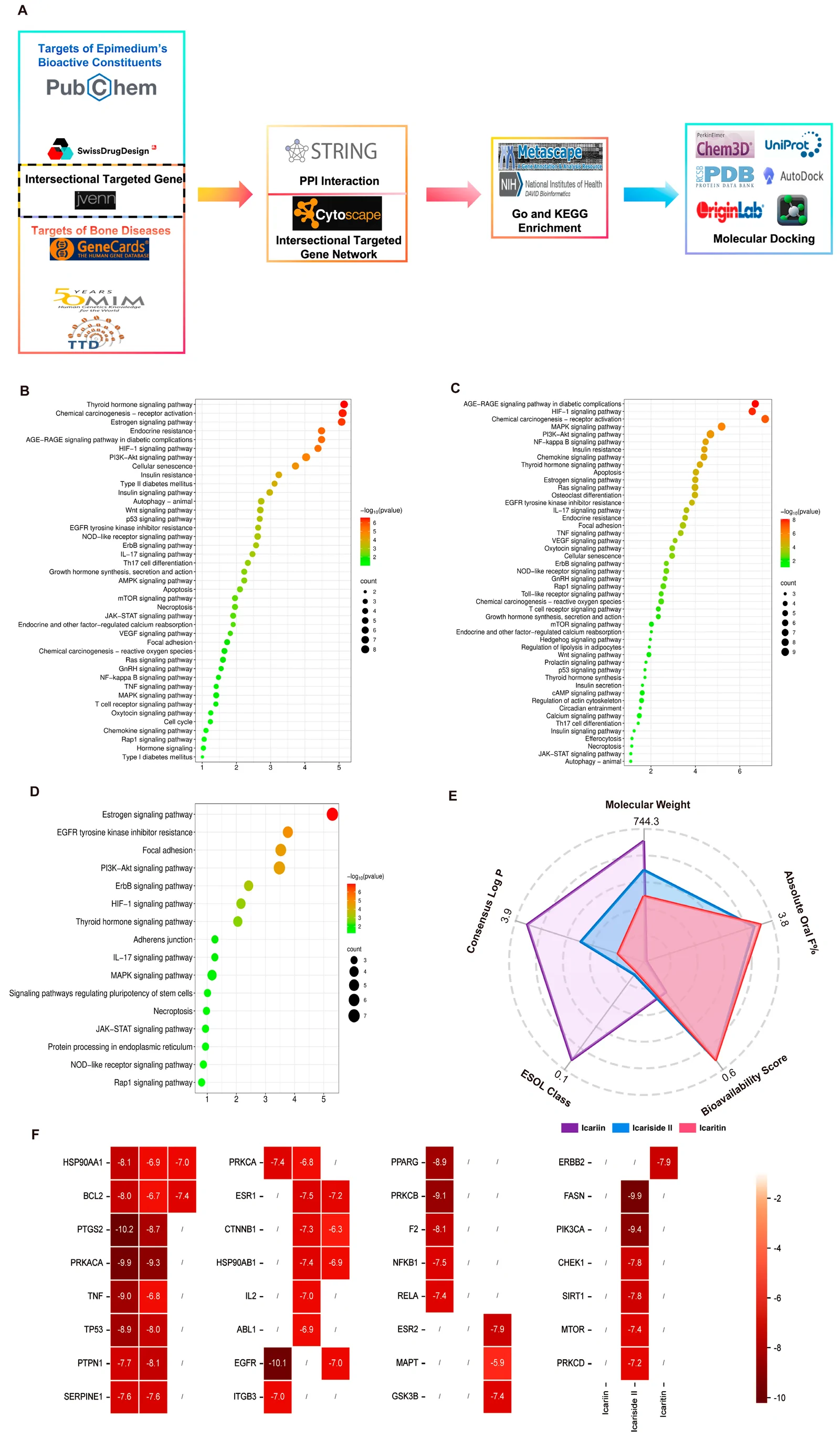
Schematic of the network pharmacology paradigm: (**A**) A flowchart showing the network pharmacological workflow for the investigation of the 53 bone diseases’ healing effects of three *Epimedium* bioactive constituents. (**B**–**D**) KEGG pathway enrichment analysis of the 53 bone diseases’ healing effects targets of icariside II, icariin, icaritin. (**E**) ADEM analysis results of icariin, icariside II, icaritin. (**F**) Heat map of molecular docking binding energy.

**Figure 5:**
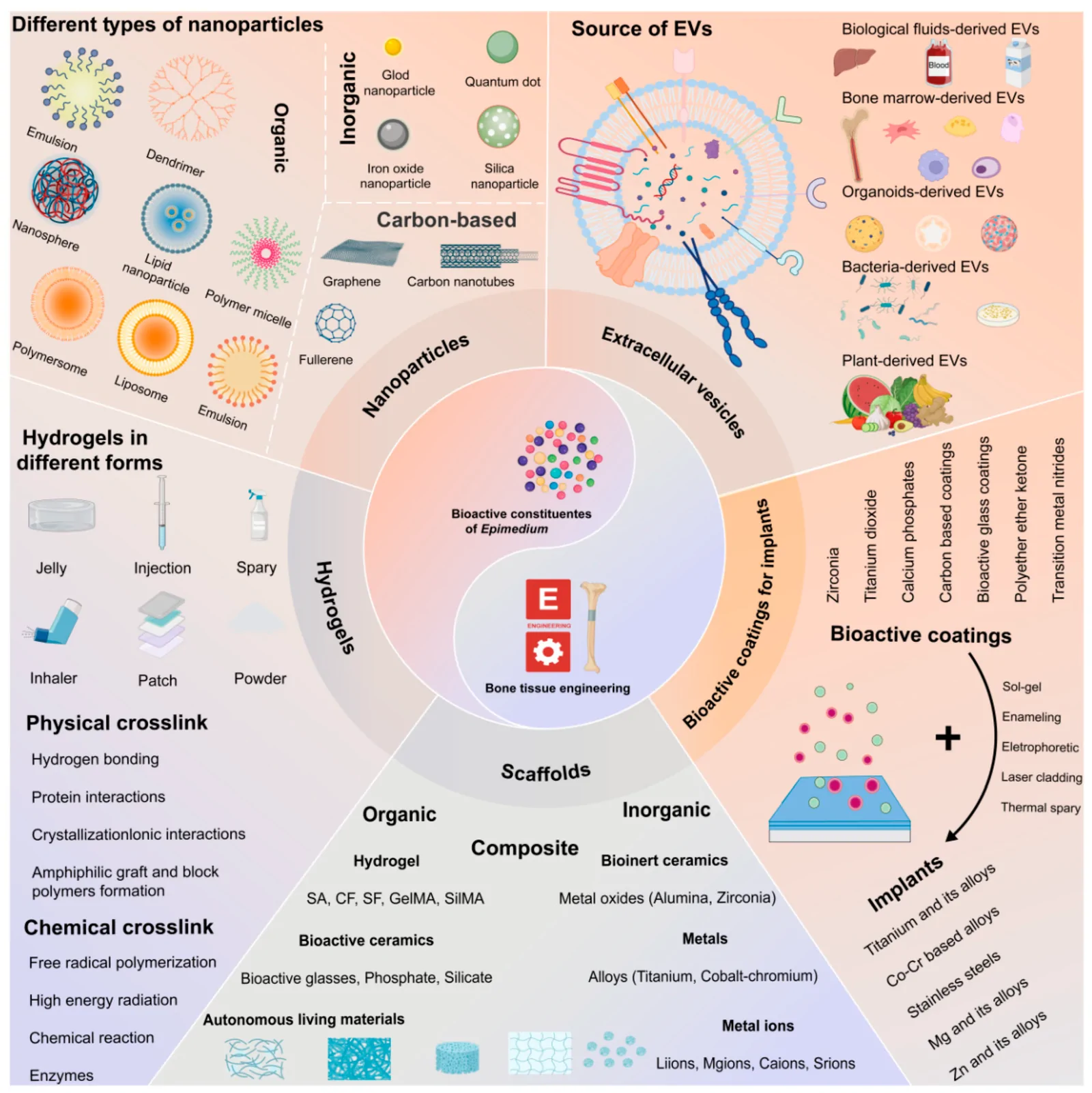
Schematic of five distinct biomaterial delivery systems: Integrating *Epimedium*-derived bioactive constituents (e.g., icariin, icaritin, etc.) with bone tissue engineering to enhance bioavailability and efficacy: (1) Hydrogels: Development of different hydrogel matrices via physical (e.g., hydrogen bonding, protein interactions, etc.) or chemical (e.g., photopolymerization, enzymatic reactions, etc.) crosslinking mechanisms to enhance drug delivery efficacy and minimize adverse effects. (2) Scaffolds: Construction of hierarchical architectures using organic polymers (e.g., hydrogel, bioactive ceramics, autonomous living materials), inorganic polymers (e.g., bioinert ceramics, metals, metal ions), or composites to provide structural support. (3) Nanoparticles: Different types of nanoparticles—such as organic systems (e.g., emulsions, dendrimers, and nanospheres), inorganic systems (e.g., gold nanoparticles and quantum dots), and carbon-based materials (e.g., graphene, silica nanoparticles, and fullerenes) have been engineered to facilitate passive targeting, enabling their extravasation into osseous defect regions. (4) Extracellular vesicles: Utilization of bio-inspired vesicles derived from varying sources including biological fluids, bone marrow, organoids, bacteria and plants for controlled drug release. (5) Bioactive coatings for implants: Functionalization of implant material surfaces through surface modification strategies (e.g., sol–gel method, enameling, etc.) applied to materials (e.g., titanium and its alloys, cobalt–chromium-based alloys, etc.), enabling the construction of bioactive coating composites that enhance the biological performance of the implant surface and more effectively promote bone defect repair.

## Data Availability

The data are provided in the article and Supplementary Materials. The network pharmacology and molecular docking analyses used publicly available databases, as detailed in Section 3.10. Additional information is available from the corresponding author upon reasonable request.

## Supplementary Materials

The following supporting information can be downloaded at: https://www.mdpi.com/article/10.3390/ijms27219579/s1.

## Abbreviations

The following abbreviations are used in this manuscript:

VEGF	Vascular Endothelial Growth Factor
Ti	Titanium
PEEK	Polyetheretherketone
MSCs	Mesenchymal Stem Cells
MAPK	Mitogen-activated Protein Kinase
NF-κB	Nuclear Factor kappa-B
RANKL	Receptor Activator of Nuclear Factor kappa-B ligand
TNF-α	Tumor Necrosis Factor-alpha
IL	Interleukin
OVX	Ovariectomy
BMSCs	Bone Marrow-derived MSCs
BMPs	Bone Morphogenetic Proteins
OPN	Osteopontin
RhoA	Ras Homolog Family Member A
ASCs	Adipose-derived Stem Cells
EphA2	Erythropoietin-producing Hepatocellular A2
ERK	Extracellular Signal-regulated Kinase
JNK	c-Jun N-terminal Kinase
CXCL12	C-X-C Motif Chemokine Ligand 12
ALP	Alkaline Phosphatase
ESR	Estrogen Receptor
ESRα	ESR alpha
PI3K	Phosphoinositide 3-Kinase
AKT	PI3K/Protein Kinase B
Smad	Drosophila Mothers Against Decapentaplegic Homolog
ROS	Reactive Oxygen Species
pGSK-3β	p-Glycogen Synthase Kinase 3β
FAK	Focal Adhesion Kinase
PPAR	Peroxisome Proliferator-activated Receptor
LPS	Lipopolysaccharide
Nrf2	Nuclear Factor Erythroid 2-related Factor 2
Saos-2 cells	Human Osteoblast-like Cells
OPG	Osteoprotegerin
Bcl-2	B-cell Lymphoma/Leukemia-2
Bax	Bcl-2-associated X Protein
mTOR	Mammalian Target of Rapamycin
AGEs	Advanced Glycation End Products
RAGE	Receptor for AGEs
hDPSCs	Human Dental Pulp Stem Cells
HO-1	Heme Oxygenase-1
HIF-1α	Hypoxia-inducible Factor-1α
TRAF6	Tumor Necrosis Factor Receptor-associated Factor 6
RANK	Receptor Activator of Nuclear Factor κB
NFATc1	Nuclear Factor of Activated T cells c1
AP-1	Activator Protein 1
AMPK	AMP-activated Protein Kinase
TLR4	Toll-like Receptor 4
uPAR	urokinase-type Plasminogen Activator Receptor
c-Fos	Proto-Oncogene c-Fos
CTSK	Cathepsin K
TRAP	Tartrate-Resistant Acid Phosphatase
IκBα	NF kappa B alpha
M-CSF	Macrophage Colony-stimulating Factor
TGF-β	Transforming Growth Factor-β
cAMP	Cyclic Adenosine Monophosphate
V-ATPase	Vacuolar H-adenosine Triphosphatase
ARE	Antioxidant Response Element
NLRP3	NOD-like Receptor Family Pyrin Domain-containing 3
ECM	Extracellular Matrix
Col	Collagen
SIRT1	Sirtuin 1
CIA	Collagen-Induced Arthritis
STAT3	Signal Transducer and Activator of Transcription 3
iNOS	inducible Nitric Oxide Synthase
NOX	Nicotinamide Adenine Dinucleotide Phosphate Oxidase
RA-FLS	Rheumatoid Arthritis Fibroblast-like Synoviocytes
3D	Three-Dimensional
NO	Nitric Oxide
eNOS	endothelial NOS
cGMP	cyclic Guanosine Monophosphate
HUVECs	Human Umbilical Vein Endothelial Cells
SAPK	Stress-activated Protein Kinase
KEGG	Kyoto Encyclopedia of Genes and Genomes
HER1	Human Epidermal Growth Factor Receptor 1
GnRH	Gonadotropin-releasing Hormone
Rap1	Ras-related Protein 1
F2	Coagulation Factor II
HSP90AA1	Heat Shock Protein 90kDa alpha A1
PRKACA	Protein Kinase cAMP-dependent Catalytic alpha
PRKCA	Protein Kinase C alpha
PTGS2	prostaglandin-endoperoxide synthase 2
PTPN1	Protein Tyrosine Phosphatase Non-Receptor Type 1
SERPINE1	Serpin Peptidase Inhibitor Clade E Member 1
TP53	Tumor Protein 53
CTNNB1	Catenin beta 1
HSP90AB1	Heat Shock Protein 90kDa alpha B1
CTS	Chitosan
HEMA	Hydroxyethyl Methacrylate
PNVCL	poly(N-vinyl caprolactam)
HA	Hyaluronic Acid
SA	Sodium Alginate
DOX	Doxorubicin
PF127	Pluronic F127
PF68	Pluronic F68
β-CD	β-cyclodextrin
PLA	Polylactic Acid
PCL	Polycaprolactone
EGCG	Epigallocatechin Gallate
SIS	Small Intestinal Submucosa
CPC	Calcium Phosphate Cement
Sr	Strontium
BCP	Biphasic Calcium Phosphate
β-TCP	β-tricalcium Phosphate
PLGA	poly (lactic-co-glycolic acid)
SAON	Steroid-associated Osteonecrosis
HFMSCs	Human Fetal Mesenchymal Stem Cells
SO	Sheep Oil
EVs	Extracellular Vesicles
sEVs	small EVs
